# Progressive oxygenation of developing leaves directs morphogenesis

**DOI:** 10.1126/sciadv.aef2430

**Published:** 2026-08-28

**Authors:** Gabriele Panicucci, Vinay Shukla, Viktoriia Voloboeva, Leonardo Jo, Kees van Kollenburg, Sara Buti, Laura Dalle Carbonare, Federico M. Giorgi, Francesco Licausi, Daan A. Weits

**Affiliations:** ^1^ Experimental and Computational Plant Development, Institute of Environment Biology, Utrecht University, Utrecht, Netherlands.; ^2^ Department of Biology, University of Oxford, Oxford, UK.; ^3^ School of Biosciences, University of Nottingham, Loughborough LE12 5RD, UK.; ^4^ Plant Stress Resilience, Institute of Environment Biology, Utrecht University, Utrecht, Netherlands.; ^5^ Department of Pharmacy and Biotechnology, University of Bologna, Bologna, Italy.

## Abstract

Oxygen availability underpins energy production in multicellular organisms, yet internal oxygen gradients arise in both plants and animals. Plants sense these variations via the plant cysteine oxidase branch of the N-degron pathway, which regulates the stability of key transcription factors. Originally linked to metabolic control, this pathway recently emerged as a development regulator. While the shoot apical meristem was shown to be hypoxic, the oxygen dynamics of organs originating from this low-oxygen niche remain unknown. Here, we show that developing leaves form a spatiotemporal oxygen gradient that is sensed through the oxygen-sensing machinery. This pathway integrates local oxygen availability to regulate leaf morphogenesis: Early hypoxia restricts cell expansion, whereas subsequent distal-to-proximal oxygenation depletes group VII ethylene response factors enabling specialized cell-fate acquisition and controlling proliferation. Our findings reveal that oxygen acts as a positional cue in normal growth, guiding developmental trajectories. This highlights opportunities to harness oxygen gradients and sensing to direct plant form and function.

## INTRODUCTION

Molecular oxygen leads a double life in plants. On the one hand, oxygen’s primary recognized contribution to plant physiology is serving as the final electron acceptor in mitochondrial aerobic energy production. On the other hand, oxygen also functions as a signaling molecule that influences the stability of key transcription factors that trigger both stress responses and developmental processes. This regulatory role is mediated by the activity of PLANT CYSTEINE OXIDASES (PCOs), which use oxygen as a cosubstrate to oxidize proteins harboring an exposed N-terminal cysteine (*1*–*3*). This posttranslational modification acts as a tag for degradation through the N-degron proteolytic pathway, de facto rendering these proteins oxygen labile (*4*). Scarcity of molecular oxygen, which leads to the stabilization of PCO substrates, can occur acutely as a result of exogenous calamitous events such as flooding or, chronically, as observed in meristematic tissues such as the shoot apical meristem (SAM) (*5*, *6*). Perturbation of this endogenous hypoxic state, for instance, through hyperoxia treatments, reduces leaf initiation rates, suggesting that hypoxia is not merely tolerated in the SAM but, instead, potentially plays an active role in maintaining meristematic function (*7*). Group VII ethylene response factors (ERFVII), little zipper 2 (ZPR2), and vernalization 2 (VRN2), oxygen-labile PCO substrates, are stable in the hypoxic meristematic niche and contribute to the regulation of root architecture, meristem activity, vernalization, and cell expansion (*7*–*10*).

While the hypoxic nature of the SAM, its maintenance, and its functional implications have been shown previously, the oxygen dynamics of organs originating from this hypoxic niche are, so far, unexplored (*11*). Leaves start their developmental track as a bulge of proliferative cells on the periphery of the hypoxic SAM. As the primordium emerges, proliferation is gradually confined to the basal portion of the leaf and eventually stops; in its place, differentiation proceeds from the apical portion of the leaf toward the base, leading to the formation of specialized cell types (*12*). The coordinated balance between proliferation and differentiation additionally shapes the final morphology of the leaf. While auxin and cytokinin hormones are known to regulate cell proliferation and expansion during leaf development, the exact mechanisms orchestrating the distal-to-proximal progression of this process remain incompletely understood (*13*, *14*).

To fulfill their roles as the primary photosynthetic organs and regulators of gas exchange, water use, and thermoregulation, leaves build several specialized structures and cell types across their development. Crucial to their role as a source organ, chlorophyll is produced and accumulated in differentiated mesophyll cells. Gas exchange, necessary for photosynthesis, is provided by the development of guard cells through the commitment of protodermal cells to the stomatal cell lineage (*15*). Mechanical resistance across the leaf surface is guaranteed by the interdigitation of epidermal cells, which, during cellular expansion, switch from isotropic to anisotropic growth creating convex and concave lobes (*16*). Trichomes, originating from dedifferentiated epidermal cells, confer an additional layer of protection against predation and increase water retention on the leaf surface (*17*, *18*).

Mature leaves exchange gasses with the surrounding atmosphere, resulting in an internal oxygen content that is close to atmospheric level (*19*). The final morphology and cellular structure of mature leaves depend, however, on processes that are initiated in the early stages of leaf development, in close proximity to the hypoxic SAM (*20*). In this work, we set out to define the dynamics of internal oxygen content during primordia emergence and to elucidate the potential role of oxygenation in the overall development of leaves.

## RESULTS

### Early leaf development occurs under hypoxic conditions, followed by progressive tip-to-base oxygenation

**Fig. 1.**
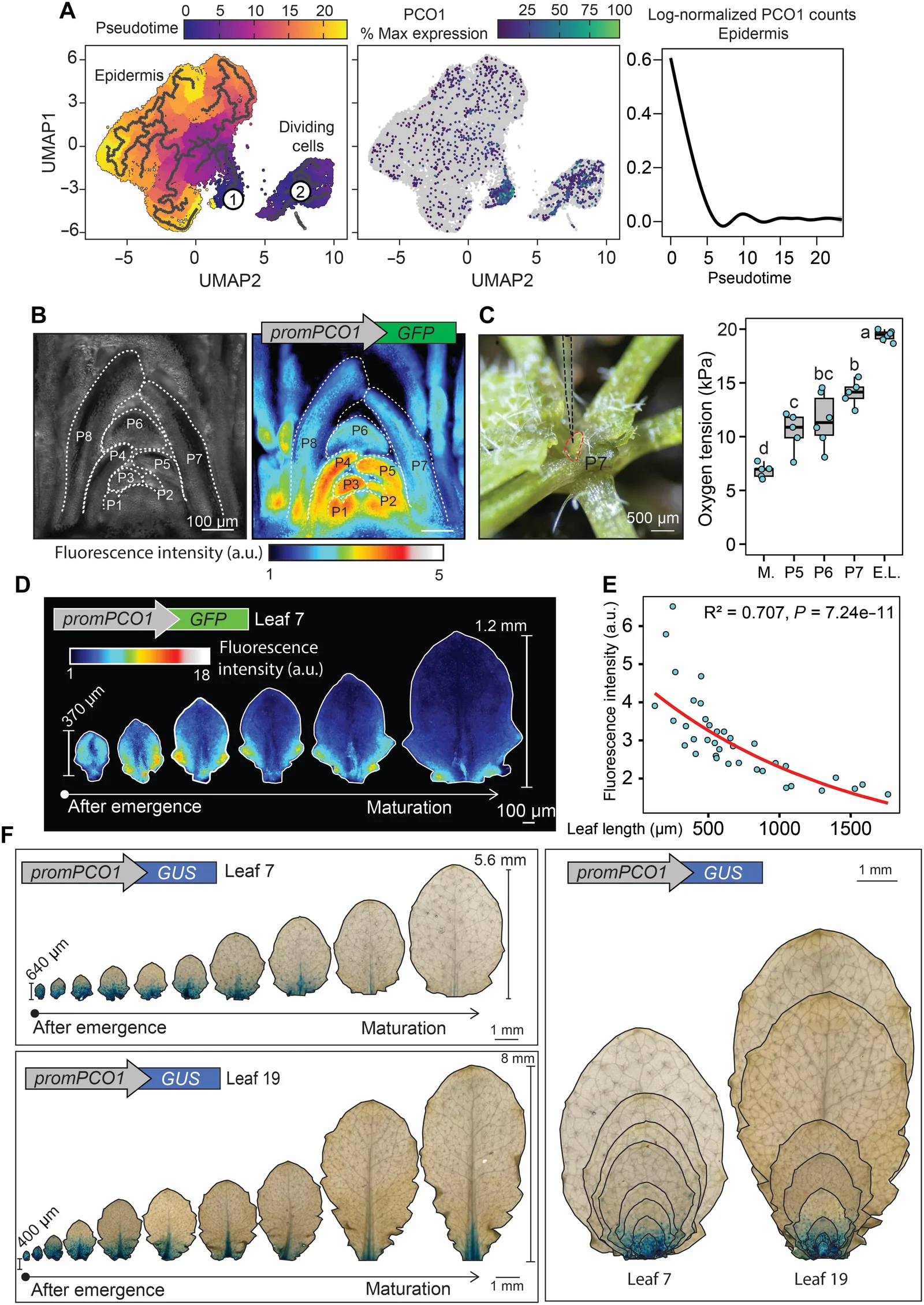
Leaves originate as a hypoxic organ and progressively oxygenate tip to base. (**A**) Uniform manifold approximation and projection (UMAP) representation of pseudotime developmental trajectory of epidermal and dividing cells isolated from 6-day-old rosettes of *A. thaliana* (*21*), colored by either pseudotime or relative expression of *PCO1*. Numbers in the white circles indicate the two separate roots for pseudotime inference in epidermal and dividing-cell clusters. Smooth generalized additive model of *PCO1* expression across pseudotime in the epidermal cluster. Shaded area reports SE. (**B**) Longitudinal vibratome sections of *A. thaliana* SAM and emerging leaf primordia. Green fluorescent protein (GFP) signal visualized with royal look-up table. Primordia (P) indicated by dashed lines and numbered starting from the most recently emerged (P1). (**C**) Oxygen microprofiling of the meristem dome (M), emerging leaf primordia (P5, P6, and P7), and fully emerged leaves (EL). Microsensor position indicated via a dashed black line, outline of P7 leaf primordia in red. a.u., arbitrary units. (**D**) Imaging of GFP signal across *promPCO1:GFP* developing leaves 7 ± 1, from emergence until maturation. Signal visualized with royal look-up table. (**E**) Quantification of GFP signal intensity in *promPCO1:GFP* developing leaves in relation to the size of their major axis. Red line shows the exponential fit obtained by linear regression of log-transformed GFP signal against length, back-transformed to the original scale. Model fit statistics are indicated on the plot [coefficient of determination (*R*^2^) and *P* value for the slope]. (**F**) GUS staining images of *promPCO1:GUS* leaves 7 and 19 across development.

Leaf primordia emerge from the SAM, a region featuring a chronically hypoxic environment (3.6 to 8.4 kPa) necessary for proper organ initiation (*7*). While mature leaves are well oxygenated, little is known as to how oxygen levels change when leaves grow out of the hypoxic niche that encloses the meristem. To investigate oxygenation dynamics during early leaf development, we first examined hypoxia-responsive genes (HRGs) in available single-nucleus transcriptomics of developing rosettes (*21*). We were able to identify a subpopulation of epidermal cells characterized by expression of several known HRGs (Fig. 1A and fig. S1A). Furthermore, the identified hypoxic signature partially colocalized with known marker of dividing cells *RPS5A* (*22*) but showed spatial segregation with marker of cell expansion *XTH22* (fig. S1B) (*23*). This suggests that the permanence of a hypoxia transcriptional signature outside of the meristematic core might be linked to the early, proliferative phases of leaf development. This is in accord with previous analysis of single-cell transcriptomes of shoots, in which a gradual decline of hypoxia markers was linked to the progression of differentiation markers (*24*). To further investigate the oxygen dynamics of emerging leaf primordia, we examined the expression of known HRG *PCO1* in SAM cross sections and confirmed that not only the meristem but also emerging leaf primordia activate low-O_2_ signaling (Fig. 1B). To bridge the gap between the observed transcriptional patterns and actual oxygen level in developing leaves, we quantified oxygen content in emerging primordia through O_2_ microprofiling (Fig. 1C). Our measurements confirmed that leaf oxygenation happens gradually during development, rather than abruptly, suggesting that low-O_2_ signaling attenuates as leaves emerge from the shoot apex. The gradual nature of oxygenation led us to question whether the entire leaf is oxygenated uniformly or rather spatially distinct regions of the leaf might experience different oxygenation dynamics. Using the HRG-based reporter line *pPCO1:GUS-GFP*, we visualized differences in low-oxygen signaling across the full developmental track of a newly formed leaf. Green fluorescent protein (GFP) fluorescence showed a clear tip-to-base deactivation of hypoxia signaling, with *pPCO1:GFP* activity being confined to the basal region in maturing leaves (Fig. 1D). GFP signal quantification across leaf length, used as a proxy for developmental stage, showed a decrease in hypoxia signaling as leaves mature (Fig. 1E). β-glucuronidase (GUS) staining of leaves 3, 7, and 19 also showed a clear pattern of distal-to-proximal oxygenation, suggesting that this gradient is consistent along the vegetative phase (Fig. 1F and fig. S2). The disappearance of hypoxia signaling seemed to correlate with the formation of mature stomata, pointing at oxygen influx through stomata as a potential driver of leaf oxygenation (fig. S3). Hypoxia in developing tissues has previously been shown to regulate developmental processes, raising the question of whether it may also act as a developmental cue during leaf formation.

### Oxygen distribution affects leaf morphology

**Fig. 2.**
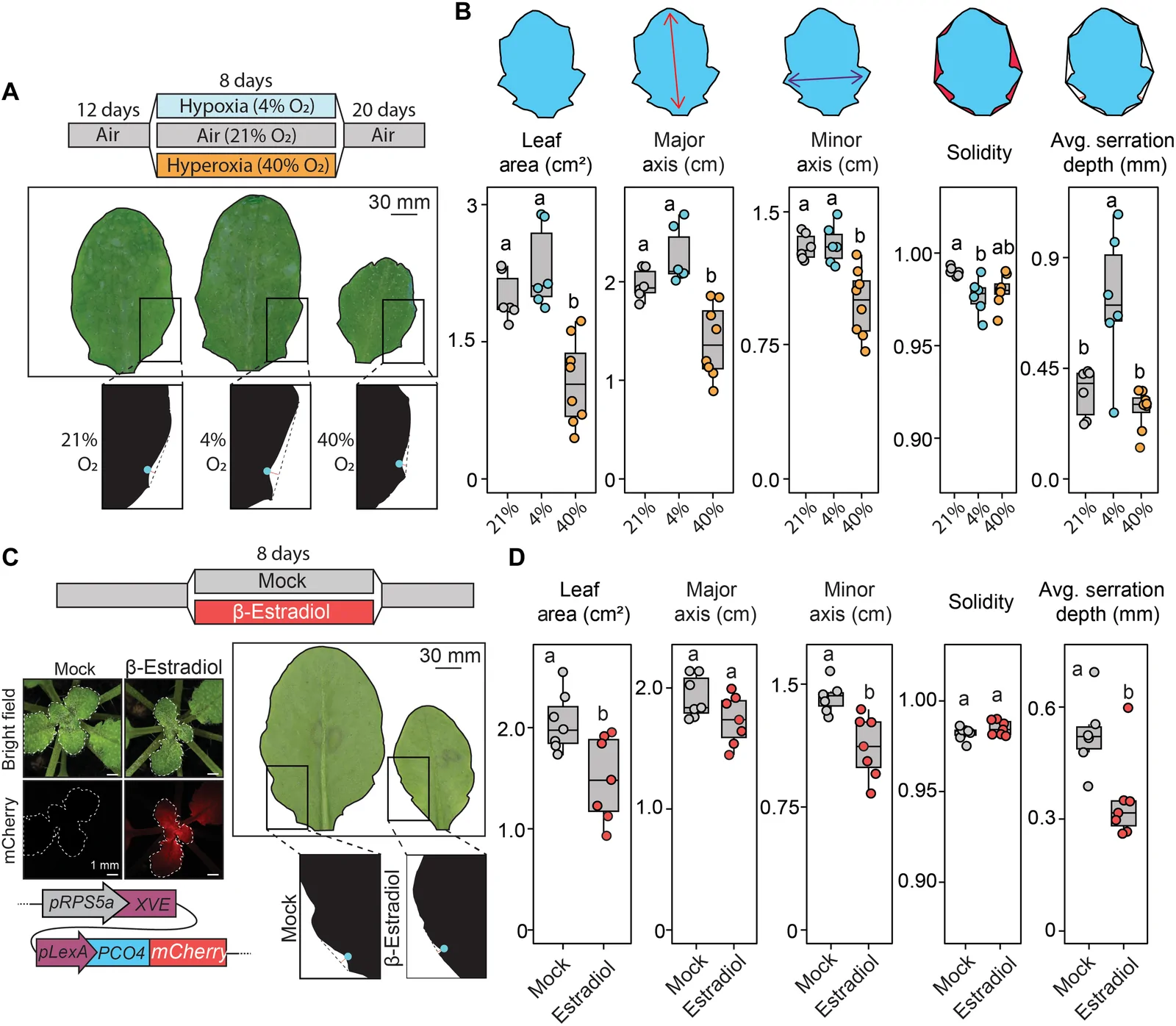
Manipulation of oxygen and oxygen signaling affects leaf shape and size. (**A**) Final shape of fully developed leaves 7 ± 1 subjected to 8 days of either 4 or 40% oxygen v/v atmospheres. Serration depth indicated on leaf silhouettes. (**B**) Morphological quantification of leaf shape and size. Solidity is calculated as the ratio between leaf area and the area of its convex hull. Average serration depth is calculated on the basis of the depth of the first two proximal serrations visible on the leaf blade. A schematic representation of each leaf trait parameter is shown above. One-way analysis of variance (ANOVA) followed by Tukey post hoc test for multiple comparisons was performed to assess statistical significance, with letters indicating statistically different groups (*P* < 0.05). (**C**) Final shape of fully developed leaves 7 ± 1 subjected to 8 days of *pRPS5a:XVE-pLEXA:PCO4-mCherry* induction. Scheme of the construct and induction visualization, via mCherry fluorescence, are also reported. Serration depth indicated on leaf silhouettes. (**D**) Morphological quantification of leaf shape and size. Student *t* test was performed to assess statistical significance. Letters indicate a statistically significant difference (*P* < 0.05).

To investigate the possibility that oxygen distribution acts as a developmental signal in leaf development, we tested whether modified oxygen atmospheres would result in altered leaf morphology. To this end, 12-day-old plants were transferred into either oxygen-depleted (4% O_2_, v/v) or oxygen-enriched (40% O_2_, v/v) artificial atmospheres (Fig. 2A). They were maintained under these conditions for 8 days and subsequently returned to ambient air (21% O_2_, v/v). Leaves that developed during the treatment completed their growth and were then scored for morphological traits. Leaves formed under hypoxic conditions were not affected in size but displayed increased complexity, characterized by deeper serrations in the proximal region (Fig. 2B). By contrast, leaves produced under hyperoxia showed a marked reduction in both major and minor axis length, which resulted in an overall reduced size. Serration depth was also diminished, although not significantly, and this likely reflected the overall reduction in leaf size (Fig. 2B). To uncouple developmental responses from potential pleiotropic effects of hyperoxia, we next opted for an attenuation of hypoxia-responses through the use of an RPS5-driven estradiol-inducible *PCO4* (*IndPCO4*) construct (Fig. 2C). *PCO4* induction during leaf development altered leaf area, primarily as a result of a reduced minor axis length (Fig. 2D). Serration depth was also reduced concomitant with a reduced leaf size. Together, these data suggest that the persistence of hypoxic conditions in lieu of progressive leaf oxygenation affects final shape to a more serrated, less solid leaf. Conversely, alleviation of low oxygen or low-oxygen signaling during the initial phases of leaf development led to stunted growth, indicating that a hypoxic environment might be needed for the early stages of leaf development in order for leaves to reach their optimal size.

### Role of the PCO branch of the N-degron pathway in leaf morphogenesis

**Fig. 3.**
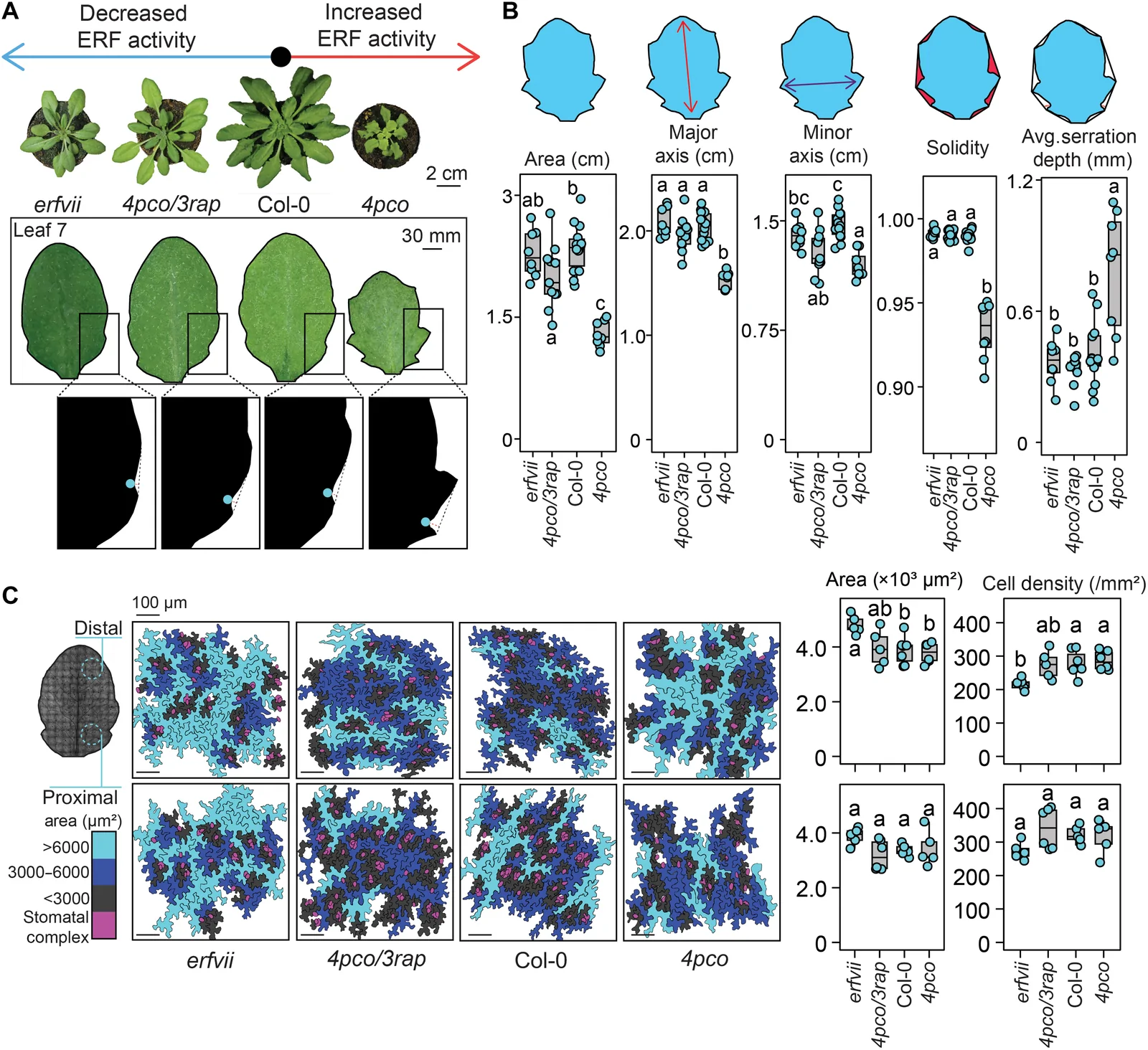
The PCO branch of the N-degron pathway affects leaf development. (**A**) Final shape of fully developed leaf 7 in different N-degron mutants. Genotypes are ordered on the basis of the predicted activity of ERFVII transcription factors in each respective mutant. Serration depth indicated on leaf silhouettes. (**B**) Morphological quantification of leaf shape and size. Solidity is calculated as the ratio between leaf area and the area of its convex hull. Average serration depth is calculated on the basis of the depth of the first two proximal serrations visible on the leaf blade. A schematic representation of each leaf trait parameter is shown above. One-way ANOVA followed by Tukey post hoc test for multiple comparisons was performed to assess statistical significance, with letters indicating statistically different groups (*P* < 0.05). (**C**) Cellular phenotype of different N-degron mutants in the distal and proximal regions of fully developed leaf 7. Segmented epidermal cells are colored on the basis of area (cyan for cells larger than 6000 μm^2^, blue for cells between 3000 and 6000 μm, and gray for cells smaller than 3000 μm). Stomatal complexes and stomatal lineage cells are indicated in magenta. Epidermal cell size and density are shown as box plots, where each point indicates the average epidermal cell size or density across one leaf. One-way ANOVA followed by Tukey post hoc test for multiple comparisons was performed to assess statistical significance, with letters indicating statistically different groups (*P* < 0.05).

Plants sense acute and chronic hypoxia via the Cys-branch of the N-degron pathway. To investigate whether this oxygen-sensing pathway plays a role in integrating oxygen distribution into leaf morphology, we next set out to examine the effects on leaf shape of different oxygen-signaling mutants that exhibit either higher or lower N-degron pathway substrates levels (Fig. 3A). We found that the *4pco* mutant, which constitutively signals hypoxia, produces distinctly more complex leaves compared with wild type (Fig. 3B). Leaves of *4pco* mutants are also smaller and rounder, but, most notably, leaf-margin analysis revealed that average serration depth was markedly enhanced, resulting in a marked decrease in solidity. Considering that the phenotype observed in *4pco* did not match the previously published phenotypes of either *ZPR2* or *VRN2* overexpression (*8*, *25*), we hypothesized that the observed *4pco* leaf complexity traits might depend on persistent stabilization of ERFVII transcription factors during leaf development. To test this hypothesis, we genetically removed *RAP2.2*, *RAP2.12*, and *RAP2.3* in the *4pco* background (*4pco3rap*) through CRISPR-Cas9 (fig. S4). The *4pco3rap* mutant displayed a slightly smaller than wild-type leaf surface area and a complete rescue of *4pco* margin-specific phenotypes, suggesting that leaf-shape–related features observed in *4pco* are dependent on ectopic stabilization of ERFVII (Fig. 3, A and B).

Next, we set out to investigate how the differences observed in final leaf morphology are linked to cellular-level phenotypes across the different mutants. To assess this, we measured the size of abaxial epidermal cells in *4pco*, *erfvii*, and *4pco3rap* fully developed leaves (Fig. 3C). We examined both distal and proximal regions, because these exhibit different oxygen dynamics during growth. The *4pco* mutant did not display differences in epidermal cell size in either the proximal or distal region, indicating that its smaller leaf size might be due to impaired proliferation. Instead, the *erfvii* mutant displayed markedly increased epidermal cell size and decreased cell number in the distal region and a minor decrease, although not significant, in cell number in the proximal region (Fig. 3C). This phenotype was mostly lost in the presence of stable and functional HRE1 and HRE2 in *4pco/3rap*. The final *erfvii* leaf shape was unaffected, indicating that, in this genotype, a compensatory reduction of cell division counteracted the increased epidermal cell size (Fig. 3, A and B). Induced expression of *PCO4* in the *RPS5* domain led to an overall smaller leaf size (Fig. 2C), which was driven by a marked reduction of epidermal cell size in the distal region of the leaf (fig. S5). This suggests that the smaller leaves of *indPCO4* and *4pco* are due to different cellular mechanisms; *PCO4* induction likely impaired cellular expansion, whereas the knockout of *4pco* led to reduced proliferation. Because *erfvii* mutants display increased epidermal cell size, this would suggest that degradation of N-degron substrates other than ERFVII was paramount to the reduced cell-size phenotype observed in *indPCO4* lines. Together, our data show that low-oxygen signaling in early stages modifies cell expansion, whereas persistence of hypoxic signaling in later stages leads to increased leaf complexity, without affecting cell expansion.

### Transcriptome changes underlying the interplay between oxygen sensing and leaf development

**Fig. 4.**
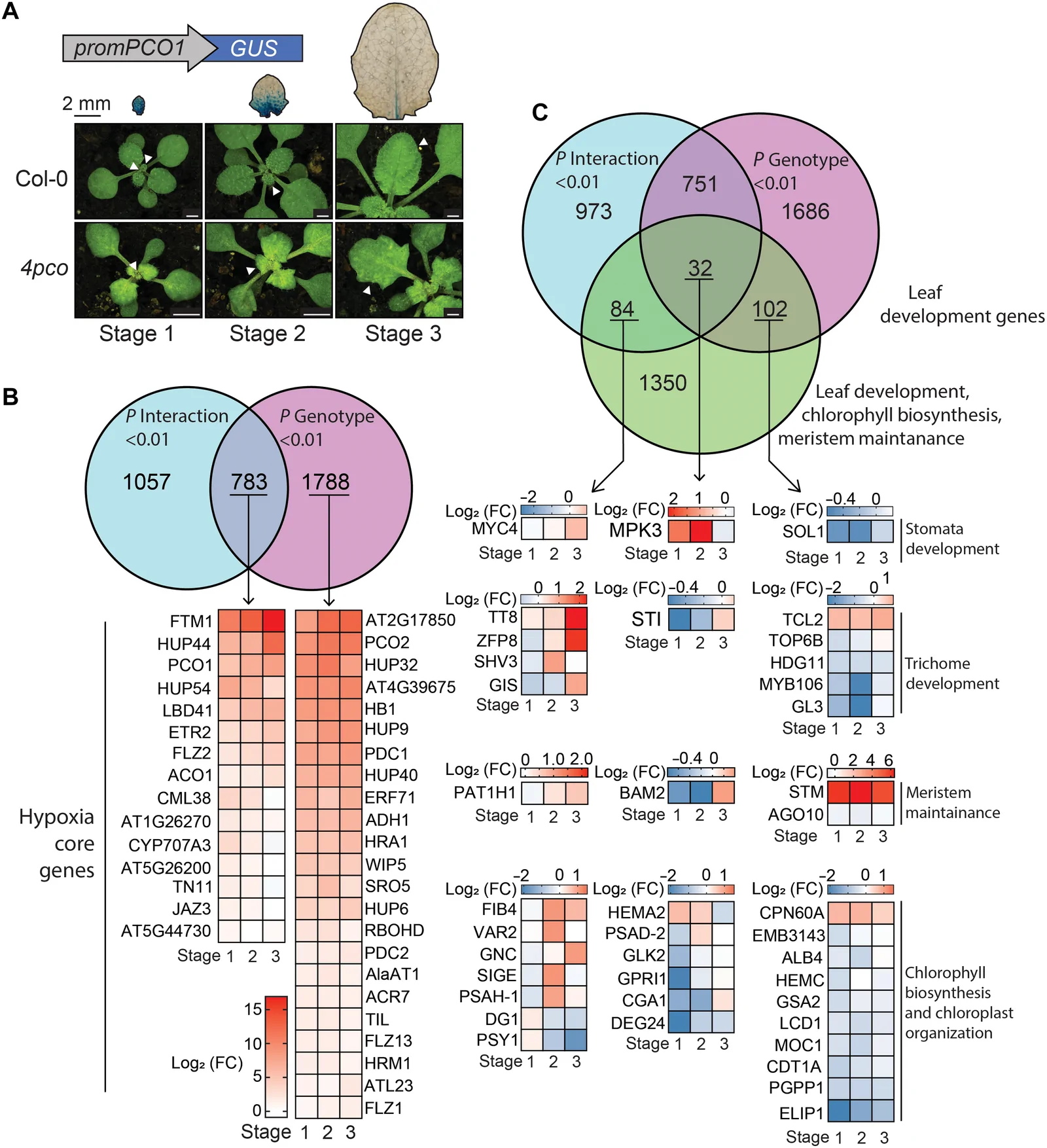
Transcriptome analysis on *4pco* reveals interplay between oxygen sensing and leaf development. (**A**) Example of sampled Col-0 and *4pco* leaves 7 ± 1 according to their hypoxic signature, inferred by activation of *promPCO1:GUS*. (**B**) List of core hypoxia genes that exhibit either a significant interaction effect or both an interaction and genotype effect. Heatmaps show fold change (FC) across stages 1 to 3 in a log_2_ scale. (**C**) Venn diagrams show the overlap between genotype, interaction effect, and leaf developmental genes. List of selected leaf development and meristem maintenance genes that exhibit either a significant interaction effect, genotype effect, or both interaction and genotype effect. Heatmaps show fold change (FC) across stages 1 to 3 in a log_2_ scale.

To understand how the dynamic oxygenation of leaves is connected at the molecular signaling level to oxygen sensing, we conducted an RNA-sequencing (RNA-seq) analysis. We compared the leaf transcriptome of the *4pco* mutant with wild-type leaves at different stages of development representing progressive stages of leaf oxygenation (Fig. 4A). Differential gene expression was analyzed using a generalized linear model with explanatory factors for genotype, developmental stage, and their interaction. We confirmed the hypoxic signature of the examined leaves by analyzing the expression pattern of the 49 known hypoxia core genes (*26*); of these, 38 were up-regulated regardless of developmental stage. Among those, 15 showed a significant interaction effect, suggesting a stage-specific layer of regulation of their expression (Fig. 4B). Furthermore, among genes that exhibited a significant interaction or genotype effect, we selected those that were associated with leaf development Gene Ontology (GO) terms (Fig. 4C). This led us to identify several misregulated genes involved in specialized cell-fate acquisition such as regulators of trichome patterning, stomata development, and meristem maintenance. In addition, we identified differential expression of several genes involved in chlorophyll biosynthesis, repair, and overall chloroplast organization (Fig. 4C). Together, the *4pco* transcriptome data suggest that constitutively-active hypoxia signaling impairs cellular differentiation of multiple leaf cell types.

### Cellular phenotyping reveals how oxygen sensing shapes cell-fate acquisition

**Fig. 5.**
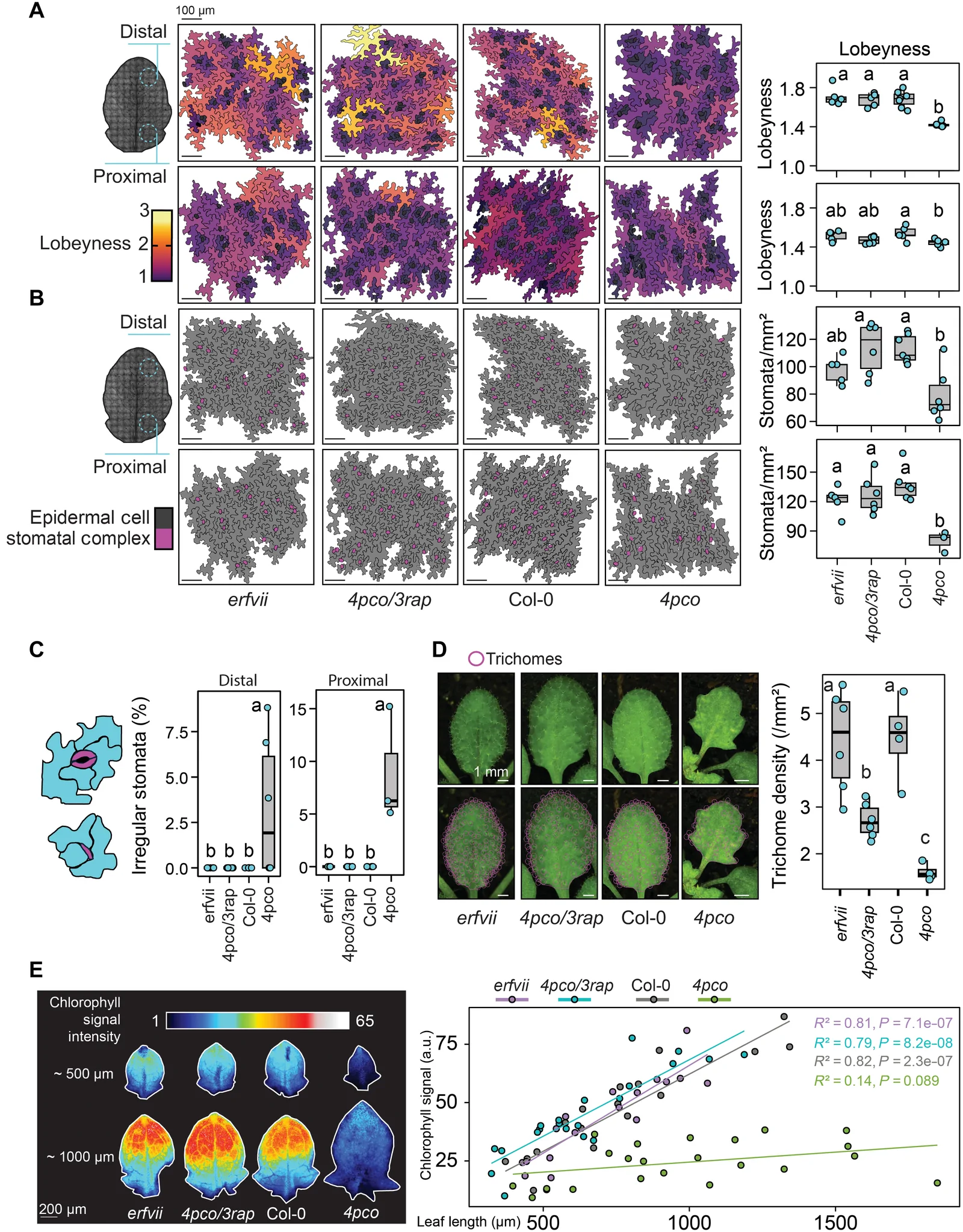
N-degron mutants are impaired in producing specialized leaf cell types. (**A**) Leaf 7 proximal and distal epidermal cells of N-degron mutants colored on the basis of their lobeyness, defined as the ratio between their perimeter and the perimeter of their convex hull. Right: Quantification of average lobeyness across genotypes. Each point represents the average lobeyness of epidermal cells across one leaf. (**B**) Stomatal complexes found in distal and proximal region of N-degron mutant leaf 7. Stomatal density was calculated as number of recognizable pairs of guard cells per square millimeter of leaf surface. For (A) and (B), the images used to indicate distal and proximal leaf sections were reused from Fig. 3C. (**C**) Percentage of irregular stomatal complexes identified in proximal and distal regions of various N-degron mutants. (**D**) Number of trichomes identified in various N-degron mutants per square millimeter of leaf surface. Image for *4pco* was reused from Fig. 4A. For (A) to (D), one-way ANOVA followed by Tukey post hoc test was used to assess statistical differences, with letters indicating statistically different groups (*P* < 0.05). (**E**) Chlorophyll autofluorescence measured in leaves 7 ± 1, visualized through royal look-up table. Right: Quantification of chlorophyll signal intensity over leaf length, with fitted linear regression for each genotype. Model fit statistics are indicated on the plot (*R*^2^ and *P* value for the slope). a.u., arbitrary units.

To assess how the apparent imbalance in cell-fate acquisition identified by our transcriptome analysis affected the formation of specialized cell types, we quantified leaf adaxial trichomes, stomatal complexes, and epidermal cell morphology in oxygen-sensing mutants. It has been previously reported that the shape of epidermal pavement cells starts isotropic and becomes progressively more complex during leaf development, exhibiting their characteristic lobey puzzle shape in mature leaves (*16*). Epidermal cells in *4pco* fail to reach the degree of complexity seen in the wild type (Fig. 5A). This suggests that the inability of *4pco* to perceive leaf oxygenation might result in simpler pavement cells. Furthermore, the number of stomatal complexes in the *4pco* distal and proximal part of the leaf was severely reduced (Fig. 5B), and a small number of these complexes exhibited irregular, single-cell stomatal precursor rather than fully formed guard cells (Fig. 5C). Considering that the number of irregular stomata only amounted up to 15% of all stomatal complexes, the differences observed in the total number of stomatal complexes in *4pco* are largely due to failure to initiate stomatal fate. The decrease both in lobeyness and in stomatal density is rescued in *4pco3rap*, suggesting that the impairment in cell-fate acquisition is dependent on ERFVII stabilization (Fig. 5, A and B). In addition, trichome density was found to be reduced in *4pco* (Fig. 5D) and partially rescued in *4pco3rap*, providing evidence of another specialized cell type that is affected by constitutively-active low-oxygen signaling. Last, altered chlorophyll-associated transcripts prompted us to investigate its biosynthesis in developing leaves. Wild-type leaves display an inverse correlation between hypoxia signaling and chlorophyll fluorescence, suggesting antagonistic regulation (Fig. 1, D and E; and fig. S6, A and B). This is supported by *4pco* chlorophyll fluorescence quantification, which was found to be lower than that of wild-type leaves across development (Fig. 5E). Collectively, cellular phenotyping supports a model in which disrupting oxygen sensing impairs both the acquisition of specialized cell types and chlorophyll biosynthesis.

### Molecular mechanisms underpinning cell-fate acquisition are targets of oxygen sensing

**Fig. 6.**
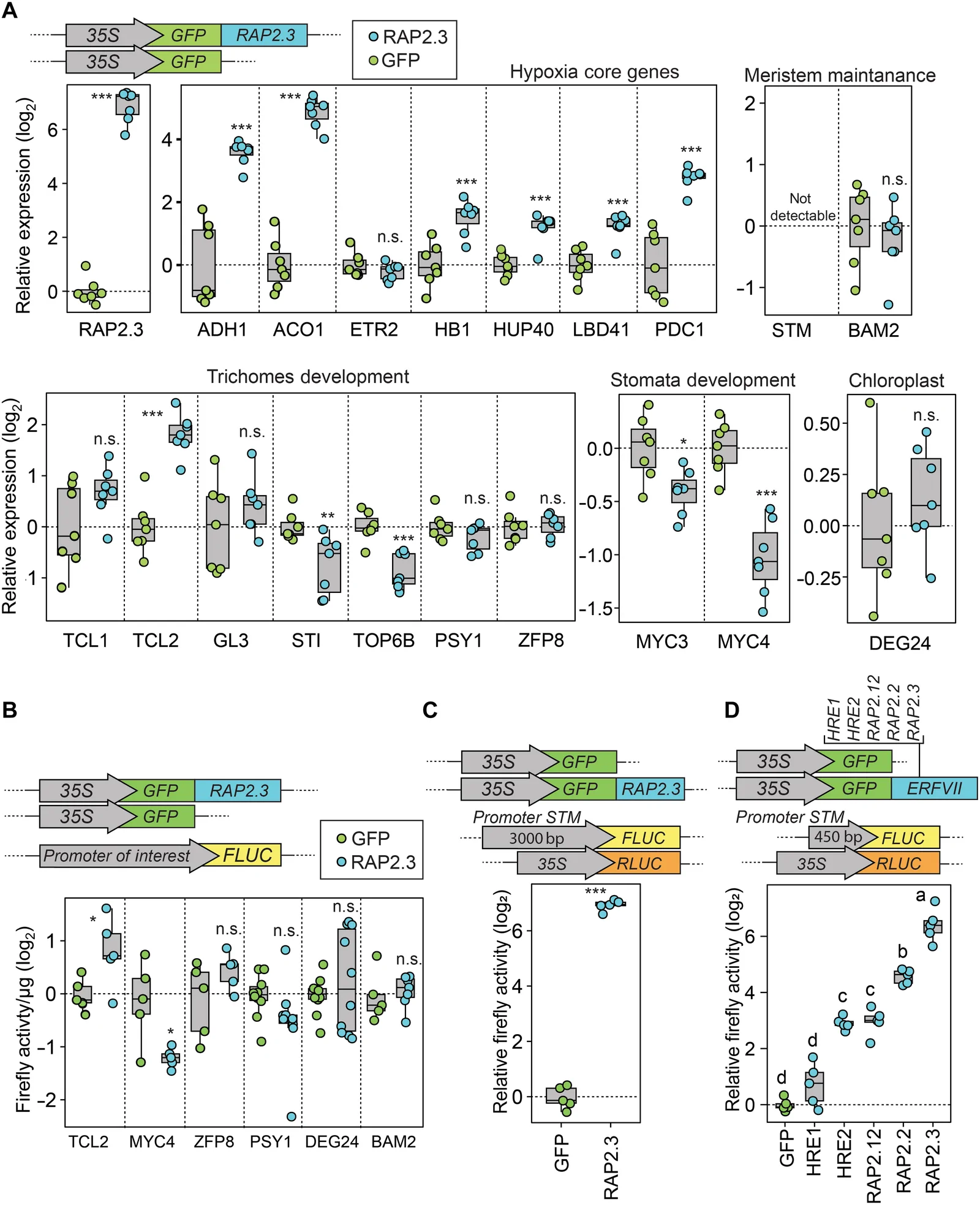
Molecular mechanisms underpinning cellular leaf traits are targets of oxygen sensing. (**A**) Reverse transcription quantitative polymerase chain reaction (RT-qPCR) on mesophyll protoplasts transformed with either *35S:GFP* (green) or *35S:GFP-RAP2.3* (cyan). Tested genes are grouped by the process they are associated with. Plots show relative expression on a log_2_ scale normalized to the relative expression of the *35S:GFP* protoplasts. (**B**) Transactivation assay testing the ability of GFP-RAP2.3 to activate or repress the promoter regions of candidate leaf development genes. Plots shows Firefly luciferase (FLUC) activity normalized on total micrograms of protein. (**C**) Transactivation assay on the STM promoter region to test the ability of GFP-RAP2.3 to induce *promSTM*. Plot shows relative Firefly luciferase activity normalized to 35S:*Renilla* luciferase (RLUC). (**D**) Ability of the different *A. thaliana* ERFVII to transactivate the 450–base pair (bp) terminal region of the *STM* promoter. Plot shows relative Firefly luciferase activity normalized to 35S:*Renilla* luciferase (RLUC). In (A) to (C), statistical differences are tested via Student or Welsh *t* test, with asterisks indicating statically different groups (*P < 0.05, **P < 0.01, and ***P < 0.001). In (D), statistical differences are assayed via one-way ANOVA and Tukey post hoc test for multiple comparisons, with letters indicating statistically different groups (*P* < 0.05). n.s., not significant.

Next, we investigated how oxygen-sensing mechanisms link to developmental genes previously identified in our RNA-seq analysis. Because the knockout of *RAP2.2*, *RAP2.3*, and *RAP2.12* in *4pco3rap* was largely sufficient to rescue the *4pco* phenotype, we reasoned that genes required for cell-fate acquisition during leaf development might be regulated through ERFVII. To test this hypothesis, we measured the expression of previously identified *4pco*-misregulated genes in isolated mesophyll protoplasts transformed with constitutive, stable *RAP2.3*. First, the induction of several hypoxia core genes attested that the introduction of RAP2.3 was sufficient to activate hypoxic responses in isolated protoplasts (Fig. 6A). In line with their differential expression in *4pco*, we detected transcriptional activation of trichome-formation inhibitor *TRICHOMELESS2* (*TCL2*) and transcriptional repression of positive regulators of trichome formation *STICHEL* (*STI*) and *TOPOISOMERASE6B* (*TOP6B*). Trichome regulators *TRICHOMELESS1* (*TCL1*), *GLABRA3* (*GL3*), and *ZINC-FINGER-PROTEIN8* (*ZFP8*) were not differentially expressed in *RAP2.3*-transformed protoplasts. Unexpectedly, negative regulators of stomatal development *MYC4* and its close relative *MYC3* were repressed by *RAP2.3*, contrary to the mild induction of *MYC4* in the *4pco* transcriptome. The expression of meristem regulator *BARELY-ANY-MERISTEM* (*BAM2*) and ribulose-1,5-bisphosphate carboxylase/oxygenase subunit *DEG24* was unchanged following RAP2.3 transformation (Fig. 6A). Specific activation and repression of the *TCL2* and *MYC3* promoter regions, respectively, was additionally confirmed through transactivation assays using RAP2.3 (Fig. 6B), whereas significant promoter activation in the presence of RAP2.3 was not detected for *ZFP8*, *DEG24*, *PSY1*, and *BAM2.* Although we were unable to detect *SHOOT-MERISTEMLESS* (*STM*) transcripts in either control or *RAP2.3* transformed mesophyll protoplasts, likely due to strong epigenetic silencing of STM in developed leaves (*27*), transactivation assays revealed that RAP2.3 could activate the *STM* promoter when provided on an exogenous vector (Fig. 6C). Further promoter dissection study of *pSTM* revealed that a 450–base pair region upstream of the translation start site is sufficient for its activation by both RAP2.3 and RAP2.12 (fig. S7). In addition, HRE2 and RAP2.2, but not HRE1, were able to activate this promoter region (Fig. 6D).

### Early transcriptomic and cellular phenotyping reveals a role for oxygen signaling in modulating cell division

**Fig. 7.**
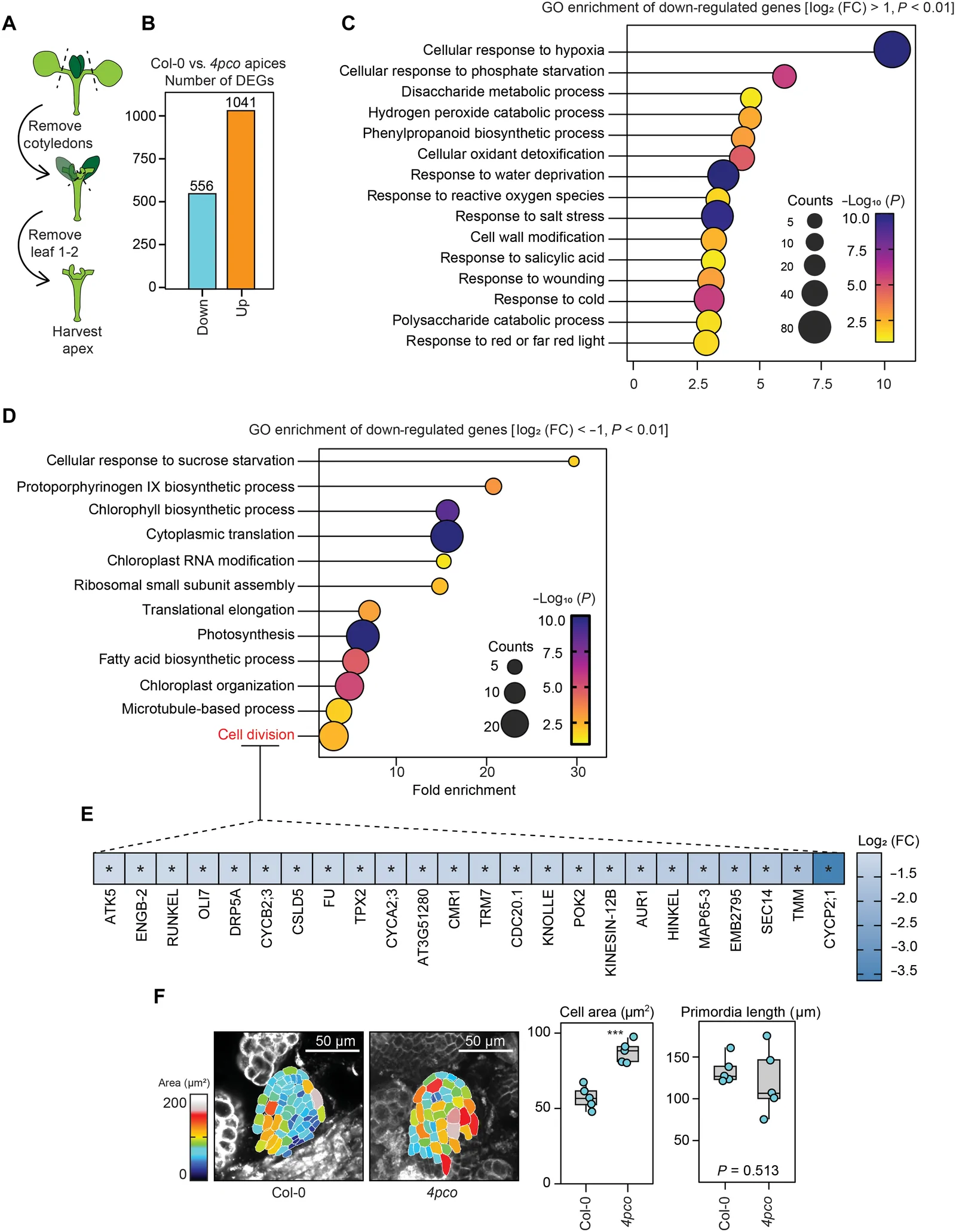
Transcriptome analysis on *4pco* primordia reveals a role for O_2_ signaling in regulating cell division. (**A**) Diagram illustrating harvesting procedure for transcriptome isolation from leaf primordia and SAMs. (**B**) Number of differentially expressed genes in *4pco* compared with those in wild-type apices. (**C**) Gene Ontology (GO) enrichment analysis of genes with at least a two-fold up-regulation in 4pco, ordered by magnitude of enrichment. Bubble size represents amount of genes detected in each category; color represents the statistical significance of their enrichment. (**D**) Same as (C), but focused on genes with at least a 0.5-fold reduction in expression. (**E**) List of genes identified in the cell-division GO category (GO:0051301), colored on the basis of their expression in *4pco* compared with that in wild type. FC, fold change. (**F**) Primordia imaging using FM4-64 cell wall staining and quantification of average cell area and leaf length. Statistical differences were assayed via Welsh *t* test, with asterisks indicating statically different groups (*P < 0.05 and ***P < 0.001).

Cellular phenotyping and transcriptional regulation revealed a role for progressive oxygenation in cellular differentiation to yield specialized leaf cell types. Conversely, the potential impact of hypoxia signaling on cell proliferation remains to be explored. A failure to switch of hypoxia signaling during leaf development in *4pco* mutants caused a marked reduction in overall leaf size, whereas epidermal cell size was indistinguishable from that of wild-type leaves (Fig. 3). This infers that the smaller *4pco* leaves comprise fewer cells, pointing to defects in cell division during early leaf development. To investigate this possibility, we sequenced the transcriptome of 1-week-old *4pco* apices, containing the SAM and emerging leaf primordia after removal of cotyledons and leaves 1 and 2 (Fig. 7A). We identified 1041 and 556 up-regulated and down-regulated genes in *4pco* apices, respectively (Fig. 7B). As expected, GO enrichment analysis of the up-regulated genes showed a marked abundance of transcripts involved in cellular response to hypoxia (Fig. 7C). Among the down-regulated genes, GO enrichment analysis is dominated by terms related to chlorophyll biosynthesis and chloroplast function, consistent with the chlorophyll-defective phenotype of *4pco* (Fig. 5E). Notably, genes down-regulated by 4pco were also significantly enriched for the GO term GO:0051301 (cell division), with 24 differentially expressed transcripts falling within this category (Fig. 7, D and E). In this group, *RUNKEL*, *CDSL5*, *FU*, *TPX2*, *KNOLLE*, *POK2*, *KINESIN-1B*, *HINKEL*, *AUR1*, and *MAP65-3* were previously shown to be expressed during cell division and function in microtubule assembly and positioning during division and cell-plate formation (*28*–*37*). In addition, *OLI7*, *CYCA2;3*, *CYCB2;3*, *CYCP2;1*, and *AUR1* were shown to regulate cell cycle progression (*36*, *38*–*41*). To confirm that the observed transcriptional pattern in *4pco* leads to an actual impairment in cell division during primordia development, we imaged *4pco* leaf primordia at 2 days after initiation and measured average cell size. Whereas the overall primordia size was comparable between wild type and *4pco*, cell size was markedly increased in the mutant, suggesting that strong hypoxia signaling in the early stages of leaf development leads to an overall slowing down of cell division (Fig. 7F).

### Hypoxia signaling affects both proliferation and cellular differentiation to shape the *4pco* phenotype

**Fig. 8.**
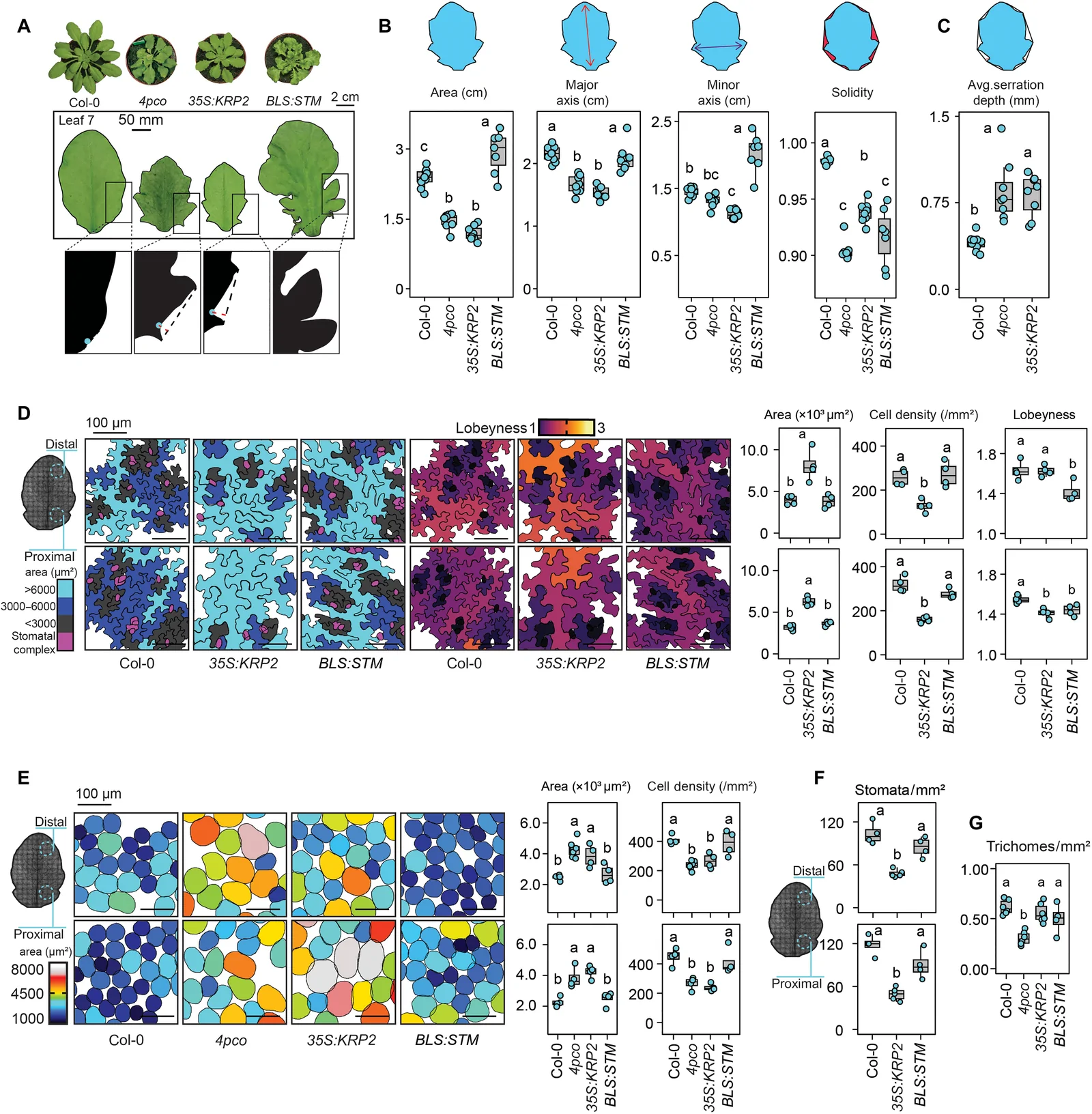
Defects in differentiation and proliferation combine downstream of impaired O_2_ signaling. (**A**) Final shape of fully developed leaf 7 in *4pco*, division-impaired *35S:KRP2* and differentiation-impaired *BLS:STM*, with corresponding whole-rosette images on top. Serration depth indicated on leaf silhouettes. (**B**) Morphological quantification of leaf shape and size. A schematic representation of each leaf trait parameter is shown above each plot. (**C**) Average serration depth calculated for genotypes that display clear serrations. (**D**) Segmentation and morphology quantification of epidermal cells in the lines presented in (A), minus *4pco* (see Figs. 3C and 5A). Cells are colored either based on area (cyan for cells larger than 6000 μm^2^, blue for cells between 3000 and 6000 μm^2^, and gray for cells smaller than 3000 μm^2^) or lobeyness [inferno look-up table (LUT)], ranging from 1 to 3). Epidermal cell size, density, and lobeyness are also shown as box plots with each point representing average measurements across one leaf. (**E**) Segmentation and size quantification of palisade mesophyll cells in the lines presented in (A). Cells are colored on the basis of area (royal LUT, ranging from 1000 to 8000 μm^2^). Mesophyll cell size and density are also shown as box plots, with each point representing average measurements across one leaf. (**F**) Quantification of stomatal density across the different genotypes. For (D) to (F), the images used to indicate distal and proximal leaf sections were reused from Fig. 3C. (**G**) Quantification of adaxial trichome density across the different genotypes. For (D) to (F), measurements are provided for both the distal and proximal regions of the leaf. For all panels showing box plots, statistical significance was assessed via one-way ANOVA followed by Tukey post hoc test for multiple comparisons, with letters indicating statistically different groups (*P* < 0.05).

**Fig. 9.**
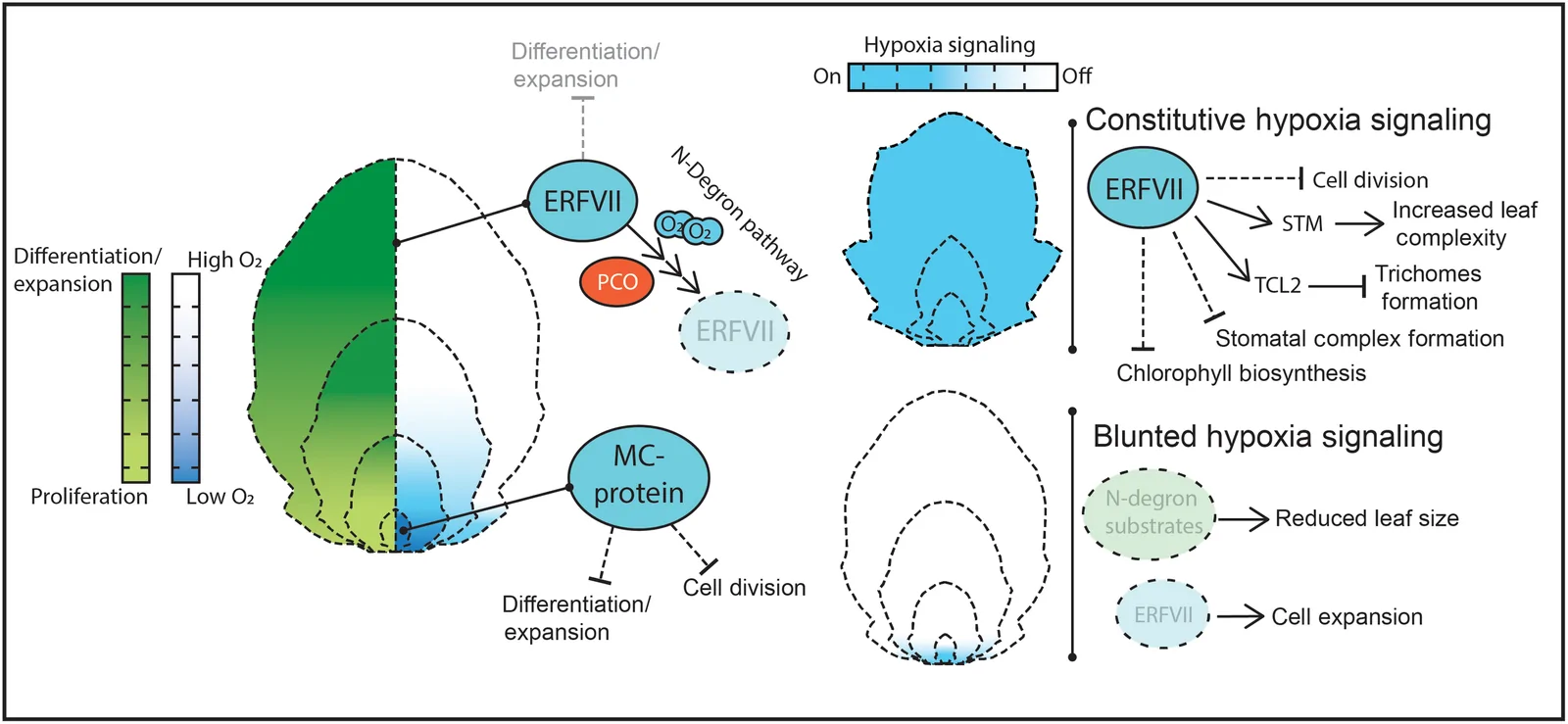
Proposed interplay of oxygen sensing and leaf development. Hypothetical model showing how progressive oxygenation during leaf development interplays with leaf morphology, cell expansion, and fate acquisition. Initial and leaf proximal hypoxia promotes ERFVII stability, regulating cell expansion and cell division. Distal-to-proximal oxygenation destabilizes ERFVII via the PCO branch of the N-degron pathway mediating cellular differentiation and expansion. Constitutive hypoxia signaling or blunting of hypoxic responses interferes with leaf development as shown on the right.

Having shown how low-O_2_ signaling affects both proliferation and differentiation across leaf development, we next set out to examine how progressive oxygenation coordinates these two processes to ultimately shape leaf morphology and cellular phenotypes. To achieve this, we compared *4pco* to established *Arabidopsis thaliana* lines with strongly impaired cell division (*35S:KRP2*) or delayed differentiation (*BLS:STM*) (Fig. 8A) (*42*–*45*). In terms of general leaf shape, *4pco* exhibited a more notable resemblance to *35S:KRP2* plants, in terms of both overall size and serration depth (Fig. 8, B and C). While *BLS:STM* exhibited a comparable decrease in leaf solidity as *4pco*, this was a result of the formation of leaflet-like structures, rather than serration depth. Different from the smaller leaves of *35S:KRP2* and *4pco*, *BLS:STM* leaves were larger, mostly as a result of lateral growth (Fig. 8B). This suggests that impaired cell division, rather than differentiation, is the major driver of the *4pco-*enhanced serration phenotype. However, the leaf epidermis of *4pco* more closely resembled that of *BLS:STM*, exhibiting a marked reduction of lobeyness uncoupled to changes in epidermal cell size in both distal and proximal regions of leaves (Figs. 3C and 8D). The decreased lobeyness of *4pco* is, therefore, likely due to an overall impairment in epidermis differentiation. Conversely, *35S:KRP2* displayed a marked increase in cell size and only exhibited a decrease in lobeyness in the proximal side, indicating that defects in cell division can also result in less lobey epidermal cells at the base of the leaf (Fig. 8D). A *35S:KRP2*-like increase in palisade mesophyll cell size was observed in *4pco*, suggesting that, in this layer, a decreased rate of cell division might have been met by a compensatory increase in cell size, as seen in *35S:KRP2* (Fig. 8E). Last, we examined the ability of all genotypes in producing specialized leaf cell types. A decrease in stomata density was observed in *35S:KRP2*, although this was most likely driven by the marked size increase of the epidermis, which, in turn, lead to an overall decreased cell density (Fig. 8, D and F). Stomata density in *BLS:STM* was comparable to wild-type leaves, whereas stomata formation was previously found to be delayed by STM during early development (*42*). This suggests that expression of *STM* under the *BLS* promoter is not sufficient to affect final stomata number (Fig. 8F). Last, no decrease in trichome density was observed for either *35S:KRP2* nor *BLS:STM*, indicating that this might be an oxygen-signaling–specific phenotype rather than a consequence of altered proliferation-to-differentiation dynamics (Fig. 8G). In summary, our findings demonstrate that low-oxygen signaling is closely connected to fundamental developmental processes in leaves, with both proliferation and differentiation being affected when leaf oxygenation is impaired during growth (Fig. 9).

## DISCUSSION

Our study revealed that a spatiotemporal oxygenation pattern in leaves shapes specific aspects of leaf morphogenesis. We showed that, on the whole-organ level, leaf primordia maintain a hypoxic status as they escape the shoot apex region rather than abruptly oxygenating once they grow out of the meristem. This hypoxic signature is maintained well beyond the very early stages of leaf development and is then progressively confined toward the proximal side of the leaf blade, creating an organ-wide gradient of oxygen signaling. The initial hypoxic phase followed by distal-proximal gradual oxygenation does not appear to be merely a by-product of leaf growth, but, rather, it appears to have been co-opted to regulate developmental processes. Prolonging the hypoxic phase through artificial depletion of available atmospheric oxygen led to an increased serration depth in fully mature leaves. Complementarily, both raising the oxygen level in the surrounding air to 40% and overexpressing *PCO4* in developing leaves led to a significant decrease in final leaf size, suggesting that the initial hypoxic phase positively affects overall growth potential. These initial experiments led us to speculate a role for molecular oxygen in leaf development, although hyperoxic treatments may also affect energy conversion and lead to reactive oxygen species (ROS) accumulation, which can affect leaf development (*46*). Additional support for a signaling function of oxygen distribution was provided by the strong leaf phenotype of the *4pco* mutant, which leads to stabilization of ERFVII and induction of *HRG*s. The rescue of increased complexity and leaf size of *4pco* by *4pco3rap* strongly points toward ectopic stabilization of ERFVII as the main contributor to the severe *4pco* phenotype. The *erfvii* mutant did not display leaf morphological changes, but did exhibit a marked increase in epidermal cell size, suggesting an additional role for ERFVII in inhibiting cellular expansion. Notably, another PCO target, VRN2, has previously been shown to regulate cell expansion and rosette size (*8*). This likely explains why overexpression of PCO4 results in smaller leaves, whereas knockout of *erfvii* does not.

Our *4pco* leaf transcriptome dataset showed misregulation of key factors that could be connected to specific leaf development processes, suggesting a broad impact of oxygen sensing on leaf development. Furthermore, these transcriptional deviations were associated with actual phenotypes in *4pco*. First, stomatal production is impaired in *4pco*. This is consistent with a down-regulation of *SOL1*, needed for stomatal lineage progression and an up-regulation of negative regulators of stomata development *MYC4* and *MPK3*. Unexpectedly, we found that *MYC4* and the closely related *MYC3* bHLH TF were actually down-regulated by RAP2.3 overexpression, indicating that their induction in *4pco* might be due to other N-degron pathway substrates. Second, trichome density is severely decreased in *4pco* and only partially rescued in the *4pco-3rap*, suggesting additional regulation independent of RAP2.2/2.3/2.12. The up-regulation of *TCL2*, a competitor of *GLABRA1* during the formation of the trichome initiation complex, is consistent with the reduction of adaxial trichomes in *4pco*. Moreover, *STI* and *TOP6B*, both necessary for trichome patterning and branching, are down-regulated in both *4pco* leaves and *RAP2.3*-overexpressing protoplasts. Third, while lobeyness of epidermal cells increases toward the distal side of the leaf in wild-type plants, it remained stably low in *4pco* leaves. We found up-regulation of *STM* in 4pco leaves and transactivation of its promoter by RAP2.3. Ectopic expression of *STM* was shown to increase leaf complexity and delay differentiation and thus the acquisition of epidermal lobeyness (Fig. 8D) (*42*). Fourth, misregulation of several chlorophyll biosynthesis and chloroplast organization genes in *4pco* matches the inability of the mutant to accumulate chlorophyll. Negative regulation of chlorophyll biosynthesis by the oxygen-sensing machinery was also demonstrated before in hypocotyls (*47*). Together, these data suggest an overall impairment in cell-fate specification and cellular differentiation when hypoxia signaling is not switched off, indicating that the signals originated during normal distal-to-proximal oxygenation of the leaf are required for acquisition of specialized cell types. Our results further indicate that multiple developmental regulators act downstream of the oxygen-sensing machinery, potentially connecting oxygen distribution directly to leaf morphogenesis. Additional studies will be required to fully elucidate the underlying molecular mechanisms. ROS molecules are oxygen-derived molecules that recently came to the fore as developmental regulators, including leaf development and meristem maintenance. The interplay of leaf oxygenation, ROS distribution, and morphogenesis represent an interesting frontier for further studies (*48*).

Impairment in differentiation alone is, however, not sufficient to explain the phenotype observed in *4pco*. Differentiation delays obtained via either ectopic expression of *STM* or gain-of-function mutations of *miR319* lead to increased overall leaf size (Fig. 8D) (*42*, *49*), whereas *4pco* mutants instead exhibited a reduction in leaf surface area. Our transcriptome data on *4pco* apices and cellular phenotyping of their leaf primordia explained this apparent dichotomy by indicating an additional effect of low-O_2_ signaling in inhibiting cell division. To understand how inhibition of both cell division and differentiation might intertwine along leaf development, we compared the *4pco* mutant to previously established *35S:KRP2* and *BLS:STM* transgenic lines. Our data indicated that *4pco* combined specific phenotypic aspects of these lines, sharing general leaf morphology with division-impaired *35S:KRP2* and epidermal structure with differentiation-impaired *BLS:STM.* These reported similarities and the fact that neither of these lines is able to phenocopy *4pco* strengthen the notion that constitutive oxygen signaling may negatively affect both proliferation and differentiation, promoting a slow-cycling, meristem-like state to developing leaf cells. This is further evidenced by the detection of ectopic expression of meristem marker *STM* in developed *4pco* leaves and the down-regulation of 24 cell cycle genes in *4pco* leaf primordia. This mimics the mechanism of HYPOXIA-INDUCIBLE FACTOR1α in promoting a quiescent state in animal stem cell niches, by inhibiting both proliferation and differentiation, hinting at the convergent evolution of a signaling role of low oxygen in these processes (*50*, *51*).

In conclusion, we show that leaves initiate in hypoxia and progressively become oxygenated from tip to base as they leave the shoot apex. Permanence of hypoxia during leaf development promotes increased leaf complexity, inhibits the acquisition of specialized cell types, and regulates rates of cell division during early development. The initial hypoxic state instead appears to be involved in regulation of cell expansion, involving ERFVII (Fig. 3) and VRN2 (*8*). Leaves were also previously shown to experience cyclic fluctuations in oxygen content, due to cellular respiration at night and photosynthesis during the day (*52*). Although our measurements were conducted in the middle of the day, it remains to be determined whether similar fluctuations also occur during the early leaf stages examined here, before the establishment of a functional photosynthetic apparatus. A more complex leaf shape has been shown to increase gas exchange in plant species that display heterophylly (*53*). For example, in *Hygrophila difformis* and *Rorippa aquatica*, heterophylly is mediated through a delay in differentiation by persistent *STM* expression, which we also observed in the leaves of *4pco* (*54*, *55*). It is tempting to speculate that the increased leaf complexity induced by persistent hypoxia during leaf development in *A. thaliana* is mechanistically reminiscent of more sophisticated heterophyllous adaptations of aquatic and amphibious plants to their wet environments. Hence, while here we found that oxygen distribution acts as an endogenous cue to regulate leaf morphology, this also lays the groundwork to investigate how environmental low oxygen may act as a signaling factor influencing leaf shape.

## MATERIALS AND METHODS

### Plant materials

*A. thaliana* Columbia-0 seeds were used as a wild-type ecotype. The *pPCO1:GUS-GFP* line and the *4pco* were previously described (*2*). The *4pco/3rap* lines used in this study were newly generated through CRISPR-Cas9 gene-editing *4pco* background. The *RPS5a:XVE/pLexA:PCO4-mCherry* was generated as described in the cloning section. The *35SK:KRP2* and *BLS:STM* lines were previously described (*42*, *45*).

### Plants growth conditions

All plants used in this study were grown on soil in short day conditions (8-hour light, 16-hour darkness, 21°C, 70% RH, light intensity at 200 μmol/m^2^ per second). Seeds were vernalized for 7 days at 4°C before growth.

### Pseudotime analysis

Single-nucleus transcriptomic data were collected and described by Guo *et al.* (*21*). In brief, nuclei were isolated from 6-day-old sterile seedlings and processed for single-nucleus RNA-seq. From this dataset, we extracted epidermal and dividing-cell nuclei for downstream pseudotime analysis (*56*). Raw unique molecular identifier (UMI) count matrices were processed using the Monocle3 package in R (*57*). Library-size normalization, log transformation, and principal components analysis (PCA) were performed using the preprocess_cds() function. The first 50 principal components were retained for downstream analysis. The normalized data were embedded into uniform manifold approximation and projection (UMAP) space using reduce_dimension(), and cells were grouped using Monocle3’s Leiden community-detection algorithm. A principal graph was learned across the UMAP using learn_graph() to infer developmental trajectories. Pseudotime was rooted on the basis of the expression of cell-division markers and HRGs within the epidermal and dividing-cell clusters.

### Vibratome sectioning of SAMs

Mature leaves from *A. thaliana* 4-week-old plants were removed to isolate the shoot apex. Samples were then embedded in 4% agarose and sectioned longitudinally using a Leica VT1000S vibratome. Average sections were 120 μm thick.

### Microsensor profiling

Oxygen microprofiling was conducted on emerging leaves of 4-week-old *Arabidopsis* plants using a Clark-type microsensor with a 10-μm tip (Unisense). The sensor was linked to a picoampere meter (Unisense) and mounted on a motorized micromanipulator (MM33, Unisense). Measurements were taken in 10-μm steps, beginning outside the leaf surface and advancing until the desired tissue region was reached. Before use, the microsensor was calibrated under two reference conditions: 0% O_2_, achieved with a sodium ascorbate solution (50 ml of sodium ascorbate in 50 ml of 0.1 M NaOH), and 21% O_2_, obtained by equilibrating medium with air. A dissection microscope on a boom stand (Zeiss Stemi 305) was used to precisely position the probe above the tissue. All profiling experiments were carried out at 20°C under low light.

### Histological staining

Developing leaves of *pPCO1:GUS-GFP* were separated from the meristem with a sharp razorblade and used for histochemical GUS staining. Leaves were fixed for 1 hour in 90% acetone on ice, washed twice with water, and immersed in GUS staining solution [100 mM buffer phosphate, 0.1% Triton X-100, 10 mM EDTA (pH 8), 0.5 mM potassium ferrocyanide, 0.5 mM potassium ferricyanide, and 200 mM X-Gluc] for 4 hours. Leaves were then cleared in several washes of 70% (v/v) ethanol and imaged using a Leica M205 FCA stereomicroscope equipped with a Leica dfc7000t camera.

### GFP and chlorophyll imaging and quantification

Excised developing leaves were imaged using a Zeiss Apotome 3 fluorescence microscope with a band-pass filter for GFP (excitation, 450 to 490; and emission, 500 to 550) and chlorophyll (excitation, 625 to 655; and emission, 665 to 715). Signal intensity across the leaf blade was quantified in Fiji. To improve visualization, the royal look-up table was applied on a set of representative images in Figs. 1C and 5E.

### Controlled gas treatments

Plants were exposed to low-oxygen or high-oxygen conditions inside plexiglass chambers that were continuously supplied with a humidified gas mixture of oxygen and nitrogen, at the ratios specified in Results. CO_2_ (440 parts per million) was added to prevent carbon limitation. During hypoxia treatments, the chambers were kept in under normal light conditions to avoid dark responses. Control plants were kept in the light for the same duration and flushed with air (21% O_2_, v/v).

### Construct design and induction in plants

The *pRPS5A:XVE-pLEXA:PCO4-mCherry* was generated using Green Gate cloning technology (*58*). The *PCO4* coding sequence was cloned from Col-0 cDNA without its endogenous STOP codon and placed upstream of a *mCherry* fluorescent protein and downstream of a *LexA* promoter in a *pGGN000* intermediate plasmid. A *pRPS5-XVE* module was assembled in a *pGGM000* intermediate module. The two intermediate modules were then combined in a *pGGZ001* to form a single construct with two transcriptional units and a hygromycin plant resistance. The construct was induced by spraying soil-grown plants with a solution of 50 μM β-estradiol (dissolved in dimethyl sulfoxide). Plants were sprayed every 48 hours over the induction period, and activation of *PCO4* was monitored via imaging of mCherry fluorescence.

### Generation of CRISPR-Cas9 lines

CRISPR-Cas9 guide constructs were generated by assembling synthetic DNA fragments into a modular expression cassette. The cassette included a U6 promoter for RNA polymerase III–driven transcription, a glycine-tRNA sequence to facilitate the multiplexing and processing of multiple guide RNAs, the guide RNA sequences, and the conserved single guide RNA scaffold required for Cas9 recognition. Transcriptional termination was ensured by a poly-T stretch42. The entire cassette was flanked by *attB* recombination sites to enable Gateway cloning into binary destination vectors for plant transformation. Guide sequences (guide RNAs) used in this study can be found on table S1.

### Leaf phenotyping

Fully developed leaves were cut at the site of the petiole, flattened with tape against a sheet of paper, and scanned using an Epson V850 Pro scanner. Binary leaf silhouettes were generated in Fiji and imported into R using the magick and imager packages. Leaf contours were extracted from the images and resampled to a fixed number of equidistant points along the perimeter. Leaf area and perimeter were calculated directly from the extracted contours using the shoelace formula. Elliptical Fourier analysis was then applied to the resampled contours to reconstruct smooth leaf outlines for subsequent shape descriptors analysis. The convex hull of each leaf was determined using the base R function chull(), and leaf solidity was calculated as the ratio of leaf area to convex hull area. Average serration depth, representing the mean shortest distance from contour points to the convex hull, was used to quantify lobing. The first two proximal serrations were used for this calculation. PCA of the reconstructed coordinates was used to define the major and minor axes.

### Cellular phenotyping

Fully developed leaves were washed and cleared by an overnight incubation in 70% (v/v) ethanol, followed by an overnight incubation in Hoyer’s solution (chloral hydrate/water/glycerol, 8 W:2 V:1 V) (*59*).Distal and proximal regions along the main leaf axis were dissected, and the abaxial epidermis was imaged using Zeiss Apotome 3 equipped with a differential interference contrast (DIC) module. Cell contours for segmentation and convex hulls were produced in Fiji. Area and perimeter of both segmented cells and their convex hulls were computed in Fiji. Lobeyness was calculated as the ratio between the perimeter of each cell and the perimeter of their convex hull. To remove stomata and stomatal lineage cells from the calculations, only cells with an area larger than 600 μm were considered. For visualization, epidermal cells were colored on the basis of either surface area or lobeyness. For lobeyness, color gradients were assigned using the ROI Color Coder macro (*60*). Counting of stomatal complexes was also performed on the same DIC images. For visualization of cells in leaf primordia, cotyledons and leaves 1 and 2 were removed from 8-day-old seedlings. The dissected apices were stained with FM4-64 (Thermo Fisher Scientific) (0.2 mg/ml) for a total of 30 min, with the first 15 min under vacuum to facilitate dye penetration. Primordia were imaged using a Zeiss LSM-880AiryScan confocal microscope, using a 514-nm excitation laser and a detection range between 590 and 760 nm. Segmentation and size quantification were performed in Fiji. Cells were colored on the basis of size using the ROI Color Coder macro.

### Trichome quantification

Bright-field images of leaves 7 from different oxygen-sensing mutants were used for quantifying adaxial trichomes in Fiji. Images were acquired using a Leica M205 FCA stereomicroscope equipped with a Leica dfc7000t camera.

### RNA-seq preparation and analysis

For the leaf transcriptomic, leaf 7 of the relevant developmental stages was harvested from developing Col-0 and *4pco* plants. Approximately 10 leaves were pooled for each biological replicate. Total RNA was extracted with a Quick-RNA Miniprep kit from Zymo Research, according to the manufacturer’s instructions. Genomic DNA removal was performed with on-column deoxyribonuclease I (DNAse I) treatment. Both mRNA library preparation (poly-A enrichment) and sequencing were performed by Novogene. Raw sequencing quality was assessed via FastQC. Trimmed reads produced via TrimGalore (*61*) were mapped to the TAIR10 Genome using Hisat2 (*62*). Absolute counts were obtained through featureCounts (*63*). For each gene, raw counts were modeled using a generalized linear model with a negative binomial distribution. The design formula (∼Genotype + Stage + Genotype:Stage) allowed testing for baseline differences between genotypes, temporal changes within the reference genotype, and whether temporal responses differed between genotypes (interaction). Statistical significance of each coefficient was assessed using Wald tests adjusted by Benjamini-Hochberg procedure for multiple testing. An adjusted *P* value of 0.01 was used as a cutoff to identify genes with a significant genotype, developmental stage, or interaction effect (full list on table S3). To identify candidate genes to connect low-oxygen sensing and leaf development, we cross referenced the list of genes with a significant genotype or interaction effect with a list of genes associated with leaf development GO categories (full list on table S4). For the apex transcriptomic, 7-day-old 4pco apices were isolated by removing cotyledons and leaves 1and 2. Approximately 50 apices were pooled for each biological replicate. Following RNA extraction, raw sequencing quality was assessed via FastQC, and reads were aligned to the TAIR10 Genome. Differential expression analysis was performed using DESeq2, comparing a total of three biological replicates per genotype. Genes with an absolute log_2_ fold change greater than 1 were selected for GO enrichment analysis using the PANTHER Classification System (*64*).

### Protoplast isolation and transformation

Mesophyll protoplasts were isolated from leaves of 4-week-old *A. thaliana* Col-0 plants using the Tape-*Arabidopsis* Sandwich method. The abaxial epidermis was removed, and the exposed mesophyll tissue was digested for 2 hours in the dark in an enzyme solution containing 1.5% cellulase R10, 0.4% macerozyme R10, 0.4 M mannitol, 20 mM KCl, and 20 mM MES (pH 5.7). Before use, the enzyme solution was incubated at 55°C for 10 min, cooled to room temperature, and supplemented with 0.1% bovine serum albumin and 10 mM CaCl_2_. Released protoplasts were collected in 40-ml tubes and pelleted at 100*g* for 2 min at 4°C. The supernatant was removed, and protoplasts were washed once with an equal volume of W5 solution [154 mM NaCl, 125 mM CaCl_2_, 5 mM KCl, and 2 mM MES (pH 5.7)]. Cells were kept on ice, in the dark, for 20 min and then pelleted again at 100*g* for 1 min at 4°C. The supernatant was removed, and the pellet was resuspended in MES–MgCl_2_–mannitol (MMG) solution [0.4 M mannitol, 15 mM MgCl_2_, and 4 mM MES (pH 5.7)]. For transformation, 100 μl of protoplast suspension was mixed with the desired plasmids and 100 μl of polyethylene glycol (PEG) solution (prepared by dissolving 4 g of PEG4000 in 3 ml of H_2_O, 2.5 ml of 0.8 M mannitol, and 1 ml of CaCl_2_; final volume, 10 ml). After 5 min of incubation in the dark, transformation was stopped by adding 440 μl of W5 solution. Protoplasts were pelleted at 100*g* for 2 min, the supernatant was removed, and cells were resuspended in 1 ml of washing and incubation (WI) solution [0.5 M mannitol, 20 mM KCl, 4 mM MES (pH 5.7), and 10 mM glucose]. Protoplasts were incubated overnight in the dark before use in transactivation assays or RNA extraction.

### Protoplast RNA extraction and qPCR

Total RNA was extracted from transformed mesophyll protoplasts. Five independent transformation aliquots were pooled for each assayed biological replicate. RNA was extracted with a Quick-RNA Miniprep kit (Zymo Research), according to the manufacturer’s instructions. Genomic DNA was removed via on-column DNAse I digestion. Following extraction, complementary cDNA was synthesized via the RevertAid First Strand cDNA Synthesis Kit (Thermo Fisher Scientific). Reverse transcription quantitative polymerase chain reaction (RT-qPCR) was set up with SYBR Green Real-Time Master Mix and run 10 ng of cDNA. Primers used in this study can be found on table S1.

### Transactivation assays

Transactivation assays were carried out using a dual-luciferase system using *Renilla reniformis* and *Photinus pyralis* luciferases. Target promoters were cloned from genomic DNA, then cloned into *pDONR207* (Thermo Fisher Scientific) using a attB × attP (BP) reaction, and subsequently recombined into the *PGWL7* vector (*65*) using LR Clonase II (Thermo Fisher Scientific) to create the reporter construct promoter:PpLuc. A 35S:*Renilla* or total protein content was used for normalization. To test the impact of RAP2.3 on each promoter, effector plasmids were prepared by transferring the coding sequences of *RAP2.3* from *pENTR-D/TOPO* into *p2GW7* (*66*). Mesophyll protoplasts were isolated as described above and transfected with 5 μg of each plasmid. Following a 16-hour incubation in WI medium, proteins were extracted using 30 μl of 1× Passive Lysis Buffer (Promega). Luciferase activities were quantified using the Dual-Luciferase Reporter Assay kit (Promega) and a GloMax 20/20 luminometer (Promega). Total protein content was measured using a colorimetric Bradford assay.

## References

[1] M. D. White, J. J. A. G. Kamps, S. East, L. J. Taylor Kearney, E. Flashman, The plant cysteine oxidases from *Arabidopsis thaliana* are kinetically tailored to act as oxygen sensors. J. Biol. Chem. 293, 11786–11795 (2018). 29848548 10.1074/jbc.RA118.003496 PMC6066304

[2] D. A. Weits, L. Zhou, B. Giuntoli, L. D. Carbonare, S. Iacopino, L. Piccinini, L. Lombardi, V. Shukla, L. T. Bui, G. Novi, J. T. van Dongen, F. Licausi, Acquisition of hypoxia inducibility by oxygen sensing N-terminal cysteine oxidase in spermatophytes. Plant Cell Environ. 46, 322–338 (2023). 36120894 10.1111/pce.14440 PMC10092093

[3] D. A. Weits, B. Giuntoli, M. Kosmacz, S. Parlanti, H.-M. Hubberten, H. Riegler, R. Hoefgen, P. Perata, J. T. van Dongen, F. Licausi, Plant cysteine oxidases control the oxygen-dependent branch of the N-end-rule pathway. Nat. Commun. 5, 3425 (2014). 24599061 10.1038/ncomms4425 PMC3959200

[4] A. Varshavsky, N-degron pathways. Proc. Natl. Acad. Sci. U.S.A. 121, e2408697121 (2024). 39264755 10.1073/pnas.2408697121 PMC11441550

[5] D. A. Weits, J. T. van Dongen, F. Licausi, Molecular oxygen as a signaling component in plant development. New Phytol. 229, 24–35 (2021). 31943217 10.1111/nph.16424

[6] A.-L. Le Gac, T. Laux, Hypoxia is a developmental regulator in plant meristems. Mol. Plant 12, 1422–1424 (2019). 31628990 10.1016/j.molp.2019.10.004

[7] D. A. Weits, A. B. Kunkowska, N. C. W. Kamps, K. M. S. Portz, N. K. Packbier, Z. Nemec Venza, C. Gaillochet, J. U. Lohmann, O. Pedersen, J. T. van Dongen, F. Licausi, An apical hypoxic niche sets the pace of shoot meristem activity. Nature 569, 714–717 (2019). 31092919 10.1038/s41586-019-1203-6

[8] R. Osborne, A.-M. Labandera, A. J. Ryder, A. Kanali, T. Xu, O. Akintewe, M. A. Schwarze, C. D. Morgan, S. Hartman, E. Kaiserli, D. J. Gibbs, VRN2-PRC2 facilitates light-triggered repression of PIF signaling to coordinate growth in *Arabidopsis*. Dev. Cell 60, 2046–2060.e5 (2025). 40147448 10.1016/j.devcel.2025.03.001

[9] A.-M. Labandera, H. M. Tedds, M. Bailey, C. Sprigg, R. D. Etherington, O. Akintewe, G. Kalleechurn, M. J. Holdsworth, D. J. Gibbs, The PRT6 N-degron pathway restricts VERNALIZATION 2 to endogenous hypoxic niches to modulate plant development. New Phytol. 229, 126–139 (2021). 32043277 10.1111/nph.16477 PMC7754370

[10] V. Shukla, L. Lombardi, S. Iacopino, A. Pencik, O. Novak, P. Perata, B. Giuntoli, F. Licausi, Endogenous hypoxia in lateral root primordia controls root architecture by antagonizing auxin signaling in *Arabidopsis*. Mol. Plant 12, 538–551 (2019). 30641154 10.1016/j.molp.2019.01.007

[11] V. Voloboeva, B. Dequeker, L. Van Doorselaer, G. Panicucci, P. Perata, P. Verboven, B. Nicolai, D. A. Weits, The hypoxic niche enclosing the shoot apical meristem is shaped by a combination of morphological features and metabolic activity. Mol. Plant 19, 1080–1099 (2026). 41761614 10.1016/j.molp.2026.02.011 PMC13139043

[12] F. Du, C. Guan, Y. Jiao, Molecular mechanisms of leaf morphogenesis. Mol. Plant 11, 1117–1134 (2018). 29960106 10.1016/j.molp.2018.06.006

[13] W. Wu, K. Du, X. Kang, H. Wei, The diverse roles of cytokinins in regulating leaf development. Hortic. Res. 8, 118 (2021). 34059666 10.1038/s41438-021-00558-3 PMC8167137

[14] Z. Zhang, A. Runions, R. A. Mentink, D. Kierzkowski, M. Karady, B. Hashemi, P. Huijser, S. Strauss, X. Gan, K. Ljung, M. Tsiantis, A WOX/auxin biosynthesis module controls growth to shape leaf form. Curr. Biol. 30, 4857–4868.e6 (2020). 33035489 10.1016/j.cub.2020.09.037

[15] D. C. Bergmann, F. D. Sack, Stomatal development. Annu. Rev. Plant Biol. 58, 163–181 (2007). 17201685 10.1146/annurev.arplant.58.032806.104023

[16] A. Sapala, A. Runions, A.-L. Routier-Kierzkowska, M. Das Gupta, L. Hong, H. Hofhuis, S. Verger, G. Mosca, C.-B. Li, A. Hay, O. Hamant, A. H. Roeder, M. Tsiantis, P. Prusinkiewicz, R. S. Smith, Why plants make puzzle cells, and how their shape emerges. eLife 7, e32794 (2018). 29482719 10.7554/eLife.32794 PMC5841943

[17] C. P. Bickford, Ecophysiology of leaf trichomes. Funct. Plant Biol. 43, 807–814 (2016). 32480505 10.1071/FP16095

[18] D. A. Levin, The role of trichomes in plant defense. Q. Rev. Biol. 48, 3–15 (1973).

[19] M. Herzog, E. Pellegrini, O. Pedersen, A meta-analysis of plant tissue O_2_ dynamics. Funct. Plant Biol. 50, 519–531 (2023). 37160400 10.1071/FP22294

[20] T. Blein, A. Pulido, A. Vialette-Guiraud, K. Nikovics, H. Morin, A. Hay, I. E. Johansen, M. Tsiantis, P. Laufs, A conserved molecular framework for compound leaf development. Science 322, 1835–1839 (2008). 19095941 10.1126/science.1166168

[21] X. Guo, Y. Wang, C. Zhao, C. Tan, W. Yan, S. Xiang, D. Zhang, H. Zhang, M. Zhang, L. Yang, M. Yan, P. Xie, Y. Wang, L. Li, D. Fang, X. Guang, W. Shao, F. Wang, H. Wang, S. K. Sahu, M. Liu, T. Wei, Y. Peng, Y. Qiu, T. Peng, Y. Zhang, X. Ni, Z. Xu, H. Lu, Z. Li, H. Yang, E. Wang, M. Lisby, H. Liu, H. Guo, X. Xu, An *Arabidopsis* single-nucleus atlas decodes leaf senescence and nutrient allocation. Cell 188, 2856–2871.e16 (2025). 40220755 10.1016/j.cell.2025.03.024

[22] D. Weijers, M. Franke-van Dijk, R. J. Vencken, A. Quint, P. Hooykaas, R. Offringa, An *Arabidopsis* Minute-like phenotype caused by a semi-dominant mutation in a *RIBOSOMAL PROTEIN S5* gene. Development 128, 4289–4299 (2001). 11684664 10.1242/dev.128.21.4289

[23] W. Xu, M. M. Purugganan, D. H. Polisensky, D. M. Antosiewicz, S. C. Fry, J. Braam, Arabidopsis TCH4, regulated by hormones and the environment, encodes a xyloglucan endotransglycosylase. Plant Cell 7, 1555–1567 (1995). 7580251 10.1105/tpc.7.10.1555 PMC161010

[24] S. Castellana, F. Fioriti, M. Marazzini, F. Cardarelli, E. Loreti, P. Perata, P. M. Triozzi, Inferring hypoxia-responsive regulators of cell fate in plant meristems through single-cell transcriptomics. npj Sci. Plants 2, 2 (2026).

[25] S. Wenkel, J. Emery, B.-H. Hou, M. M. S. Evans, M. K. Barton, A feedback regulatory module formed by LITTLE ZIPPER and HD-ZIPIII genes. Plant Cell 19, 3379–3390 (2007). 18055602 10.1105/tpc.107.055772 PMC2174893

[26] A. Mustroph, S. C. Lee, T. Oosumi, M. E. Zanetti, H. Yang, K. Ma, A. Yaghoubi-Masihi, T. Fukao, J. Bailey-Serres, Cross-kingdom comparison of transcriptomic adjustments to low-oxygen stress highlights conserved and plant-specific responses. Plant Physiol. 152, 1484–1500 (2010). 20097791 10.1104/pp.109.151845 PMC2832244

[27] D. Schubert, L. Primavesi, A. Bishopp, G. Roberts, J. Doonan, T. Jenuwein, J. Goodrich, Silencing by plant Polycomb-group genes requires dispersed trimethylation of histone H3 at lysine 27. EMBO J. 25, 4638–4649 (2006). 16957776 10.1038/sj.emboj.7601311 PMC1590001

[28] T. Krupnova, Y.-D. Stierhof, U. Hiller, G. Strompen, S. Müller, The microtubule-associated kinase-like protein RUNKEL functions in somatic and syncytial cytokinesis. Plant J. 74, 781–791 (2013). 23451828 10.1111/tpj.12160

[29] M. Lan, Y. Zhu, A. Peaucelle, X. Zhu, Y. Liu, X. Cao, A. Gurzadyan, G. Gao, W. Cai, J. Gruel, K. T. Haas, H. Jönsson, O. Hamant, R. Wightman, E. Meyerowitz, W. Yang, CSLD5-mediated cell wall remodelling regulates tissue mechanics and shoot meristem growth. Nat. Commun. 16, 7229 (2025). 40764302 10.1038/s41467-025-62651-8 PMC12325914

[30] S. A. Oh, A. Johnson, A. Smertenko, D. Rahman, S. K. Park, P. J. Hussey, D. Twell, A divergent cellular role for the FUSED kinase family in the plant-specific cytokinetic phragmoplast. Curr. Biol. 15, 2107–2111 (2005). 16332535 10.1016/j.cub.2005.10.044

[31] J. W. Vos, L. Pieuchot, J.-L. Evrard, N. Janski, M. Bergdoll, D. de Ronde, L. H. Perez, T. Sardon, I. Vernos, A.-C. Schmit, The plant TPX2 protein regulates prospindle assembly before nuclear envelope breakdown. Plant Cell 20, 2783–2797 (2008). 18941054 10.1105/tpc.107.056796 PMC2590745

[32] M. H. Lauber, I. Waizenegger, T. Steinmann, H. Schwarz, U. Mayer, I. Hwang, W. Lukowitz, G. Jürgens, The *Arabidopsis* KNOLLE protein is a cytokinesis-specific syntaxin. J. Cell Biol. 139, 1485–1493 (1997). 9396754 10.1083/jcb.139.6.1485 PMC2132613

[33] A. Herrmann, P. Livanos, E. Lipka, A. Gadeyne, M.-T. Hauser, D. Van Damme, S. Müller, Dual localized kinesin-12 POK2 plays multiple roles during cell division and interacts with MAP65-3. EMBO Rep. 19, e46085 (2018). 30002118 10.15252/embr.201846085 PMC6123660

[34] S. A. Oh, T. Allen, G. J. Kim, A. Sidorova, M. Borg, S. K. Park, D. Twell, Arabidopsis Fused kinase and the Kinesin-12 subfamily constitute a signalling module required for phragmoplast expansion. Plant J. 72, 308–319 (2012). 22709276 10.1111/j.1365-313X.2012.05077.x

[35] G. Strompen, F. El Kasmi, S. Richter, W. Lukowitz, F. F. Assaad, G. Jürgens, U. Mayer, The *Arabidopsis HINKEL* gene encodes a kinesin-related protein involved in cytokinesis and is expressed in a cell cycle-dependent manner. Curr. Biol. 12, 153–158 (2002). 11818068 10.1016/s0960-9822(01)00655-8

[36] B. Petrovská, V. Cenklová, Ž. Pochylová, H. Kourová, A. Doskočilová, O. Plíhal, L. Binarová, P. Binarová, Plant Aurora kinases play a role in maintenance of primary meristems and control of endoreduplication. New Phytol. 193, 590–604 (2012). 22150830 10.1111/j.1469-8137.2011.03989.x

[37] C.-M. K. Ho, Y.-R. J. Lee, L. D. Kiyama, S. P. Dinesh-Kumar, B. Liu, *Arabidopsis* microtubule-associated protein MAP65-3 cross-links antiparallel microtubules toward their plus ends in the phragmoplast via its distinct C-terminal microtubule binding domain. Plant Cell 24, 2071–2085 (2012). 22570443 10.1105/tpc.111.092569 PMC3442588

[38] U. Fujikura, G. Horiguchi, M. R. Ponce, J. L. Micol, H. Tsukaya, Coordination of cell proliferation and cell expansion mediated by ribosome-related processes in the leaves of *Arabidopsis thaliana*. Plant J. 59, 499–508 (2009). 19392710 10.1111/j.1365-313X.2009.03886.x

[39] K. K. Imai, Y. Ohashi, T. Tsuge, T. Yoshizumi, M. Matsui, A. Oka, T. Aoyama, The A-type cyclin CYCA2;3 is a key regulator of ploidy levels in *Arabidopsis* endoreduplication. Plant Cell 18, 382–396 (2006). 16415207 10.1105/tpc.105.037309 PMC1356546

[40] P. Bulankova, S. Akimcheva, N. Fellner, K. Riha, Identification of *Arabidopsis* meiotic cyclins reveals functional diversification among plant cyclin genes. PLOS Genet. 9, e1003508 (2013). 23671425 10.1371/journal.pgen.1003508 PMC3649987

[41] L. Peng, A. Skylar, P. L. Chang, K. Bisova, X. Wu, CYCP2;1 integrates genetic and nutritional information to promote meristem cell division in *Arabidopsis*. Dev. Biol. 393, 160–170 (2014). 24951878 10.1016/j.ydbio.2014.06.008

[42] D. Kierzkowski, A. Runions, F. Vuolo, S. Strauss, R. Lymbouridou, A.-L. Routier-Kierzkowska, D. Wilson-Sánchez, H. Jenke, C. Galinha, G. Mosca, Z. Zhang, C. Canales, R. Dello Ioio, P. Huijser, R. S. Smith, M. Tsiantis, A growth-based framework for leaf shape development and diversity. Cell 177, 1405–1418.e17 (2019). 31130379 10.1016/j.cell.2019.05.011 PMC6548024

[43] R. Malinowski, A. Kasprzewska, A. J. Fleming, Targeted manipulation of leaf form via local growth repression. Plant J. 66, 941–952 (2011). 21418354 10.1111/j.1365-313X.2011.04559.x

[44] L. De Veylder, T. Beeckman, G. T. Beemster, L. Krols, F. Terras, I. Landrieu, E. van der Schueren, S. Maes, M. Naudts, D. Inzé, Functional analysis of cyclin-dependent kinase inhibitors of Arabidopsis. Plant Cell 13, 1653–1668 (2001). 11449057 10.1105/TPC.010087 PMC139548

[45] E. Shani, Y. Burko, L. Ben-Yaakov, Y. Berger, Z. Amsellem, A. Goldshmidt, E. Sharon, N. Ori, Stage-specific regulation of Solanum lycopersicum leaf maturation by class 1 KNOTTED1-LIKE HOMEOBOX proteins. Plant Cell 21, 3078–3092 (2009). 19820191 10.1105/tpc.109.068148 PMC2782295

[46] H. Huang, F. Ullah, D.-X. Zhou, M. Yi, Y. Zhao, Mechanisms of ROS regulation of plant development and stress responses. Front. Plant Sci. 10, 800 (2019). 31293607 10.3389/fpls.2019.00800 PMC6603150

[47] M. Abbas, G. Sharma, C. Dambire, J. Marquez, C. Alonso-Blanco, K. Proaño, M. J. Holdsworth, An oxygen-sensing mechanism for angiosperm adaptation to altitude. Nature 606, 565–569 (2022). 35650430 10.1038/s41586-022-04740-y PMC9200633

[48] J. H. Schippers, C. H. Foyer, J. T. van Dongen, Redox regulation in shoot growth, SAM maintenance and flowering. Curr. Opin. Plant Biol. 29, 121–128 (2016). 26799134 10.1016/j.pbi.2015.11.009

[49] K. R. Challa, M. Rath, U. Nath, The CIN-TCP transcription factors promote commitment to differentiation in Arabidopsis leaf pavement cells via both auxin-dependent and independent pathways. PLOS Genet. 15, e1007988 (2019). 30742619 10.1371/journal.pgen.1007988 PMC6386416

[50] M. E. Hubbi, Kshitiz, D. M. Gilkes, S. Rey, C. C. Wong, W. Luo, D.-H. Kim, C. V. Dang, A. Levchenko, G. L. Semenza, A nontranscriptional role for HIF-1α as a direct inhibitor of DNA replication. Sci. Signal. 6, ra10 (2013). 23405012 10.1126/scisignal.2003417 PMC4124626

[51] Q. Lin, Y.-J. Lee, Z. Yun, Differentiation arrest by hypoxia. J. Biol. Chem. 281, 30678–30683 (2006). 16926163 10.1074/jbc.C600120200

[52] P. M. Triozzi, L. Brunello, G. Novi, G. Ferri, F. Cardarelli, E. Loreti, M. Perales, P. Perata, Spatiotemporal oxygen dynamics in young leaves reveal cyclic hypoxia in plants. Mol. Plant 17, 377–394 (2024). 38243593 10.1016/j.molp.2024.01.006

[53] A. Leigh, M. A. Zwieniecki, F. E. Rockwell, C. K. Boyce, A. B. Nicotra, N. M. Holbrook, Structural and hydraulic correlates of heterophylly in Ginkgo biloba. New Phytol. 189, 459–470 (2011). 20880226 10.1111/j.1469-8137.2010.03476.x

[54] G. Li, J. Yang, Y. Chen, X. Zhao, Y. Chen, S. Kimura, S. Hu, H. Hou, SHOOT MERISTEMLESS participates in the heterophylly of *Hygrophila difformis* (Acanthaceae). Plant Physiol. 190, 1777–1791 (2022). 35984299 10.1093/plphys/kiac382 PMC9614456

[55] H. Nakayama, N. Nakayama, S. Seiki, M. Kojima, H. Sakakibara, N. Sinha, S. Kimura, Regulation of the KNOX-GA gene module induces heterophyllic alteration in North American lake cress. Plant Cell 26, 4733–4748 (2014). 25516600 10.1105/tpc.114.130229 PMC4311196

[56] C. Trapnell, D. Cacchiarelli, J. Grimsby, P. Pokharel, S. Li, M. Morse, N. J. Lennon, K. J. Livak, T. S. Mikkelsen, J. L. Rinn, The dynamics and regulators of cell fate decisions are revealed by pseudotemporal ordering of single cells. Nat. Biotechnol. 32, 381–386 (2014). 24658644 10.1038/nbt.2859 PMC4122333

[57] J. Cao, M. Spielmann, X. Qiu, X. Huang, D. M. Ibrahim, A. J. Hill, F. Zhang, S. Mundlos, L. Christiansen, F. J. Steemers, C. Trapnell, J. Shendure, The single-cell transcriptional landscape of mammalian organogenesis. Nature 566, 496–502 (2019). 30787437 10.1038/s41586-019-0969-x PMC6434952

[58] A. Lampropoulos, Z. Sutikovic, C. Wenzl, I. Maegele, J. U. Lohmann, J. Forner, GreenGate - A novel, versatile, and efficient cloning system for plant transgenesis. PLOS ONE 8, e83043 (2013). 24376629 10.1371/journal.pone.0083043 PMC3869738

[59] Y. Cheng, L. Cao, S. Wang, Y. Li, H. Wang, Y. Zhou, Analyses of plant leaf cell size, density and number, as well as trichome number using cell counter plugin. Bio-protocol 4, (2014).

[60] T. Ferreira, K. Miura, B. Chef, J. Eglinger. Scripts: BAR 1.1.6, version 1.1.6, Zenodo (2015); 10.5281/ZENODO.28838.

[61] F. Krueger, F. James, P. Ewels, E. Afyounian, M. Weinstein, B. Schuster-Boeckler, G. Hulselmans, Sclamons, FelixKrueger/TrimGalore: v0.6.10 - add default decompression path, Zenodo (2023); 10.5281/ZENODO.7598955.

[62] D. Kim, J. M. Paggi, C. Park, C. Bennett, S. L. Salzberg, Graph-based genome alignment and genotyping with HISAT2 and HISAT-genotype. Nat. Biotechnol. 37, 907–915 (2019). 31375807 10.1038/s41587-019-0201-4 PMC7605509

[63] Y. Liao, G. K. Smyth, W. Shi, featureCounts: An efficient general purpose program for assigning sequence reads to genomic features. Bioinformatics 30, 923–930 (2014). 24227677 10.1093/bioinformatics/btt656

[64] H. Mi, A. Muruganujan, X. Huang, D. Ebert, C. Mills, X. Guo, P. D. Thomas, Protocol Update for large-scale genome and gene function analysis with the PANTHER classification system (v.14.0). Nat. Protoc. 14, 703–721 (2019). 30804569 10.1038/s41596-019-0128-8 PMC6519457

[65] R. P. Hellens, A. C. Allan, E. N. Friel, K. Bolitho, K. Grafton, M. D. Templeton, S. Karunairetnam, A. P. Gleave, W. A. Laing, Transient expression vectors for functional genomics, quantification of promoter activity and RNA silencing in plants. Plant Methods 1, 13 (2005). 16359558 10.1186/1746-4811-1-13 PMC1334188

[66] M. Karimi, D. Inzé, A. Depicker, GATEWAY vectors for Agrobacterium-mediated plant transformation. Trends Plant Sci. 7, 193–195 (2002). 11992820 10.1016/s1360-1385(02)02251-3

