# Supplementary material for: Progressive oxygenation of developing leaves directs morphogenesis
Source: Sci Adv. 2026 Aug 28;12(35):eaef2430. doi: 10.1126/sciadv.aef2430 (PMC13524023; doi:10.1126/sciadv.aef2430)
Supplement: Supplementary file 1 [file sciadv.aef2430_sm.pdf]

Science Advances
AAAS

Supplementary Materials for

Progressive oxygenation of developing leaves directs morphogenesis

Gabriele Panicucci *et al.*

Corresponding author: Daan A. Weits, d.a.weits@uu.nl

Sci. Adv. **12**, eaef2430 (2026)
DOI: 10.1126/sciadv.aef2430

The PDF file includes:

Figs. S1 to S7
Tables S1, S2 and S4
Legends for tables S3 and S5

Other Supplementary Material for this manuscript includes the following:

Tables S3 and S5

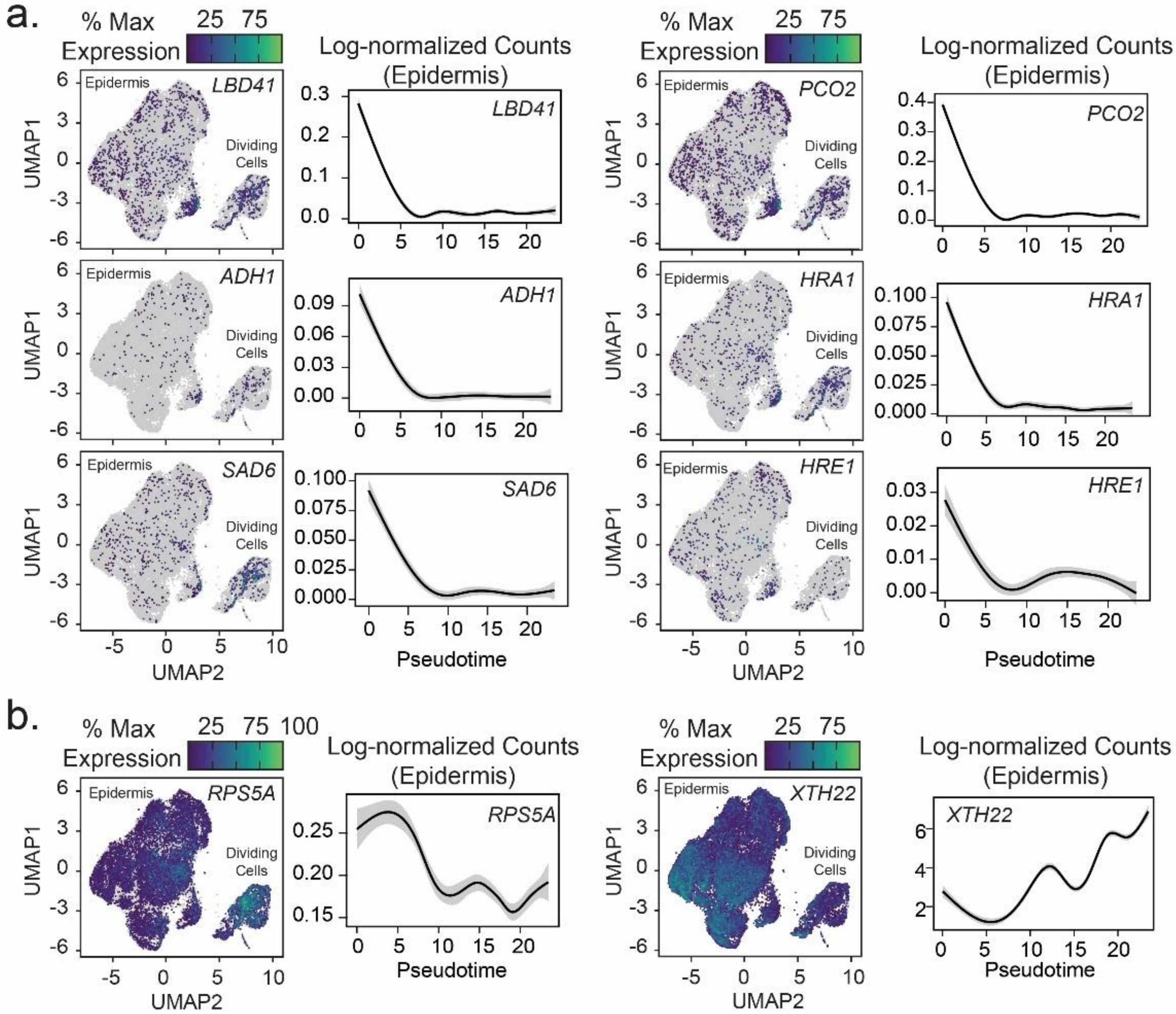

Figure S1. Expression of hypoxia, proliferation and expansion markers over pseudotime

(a) Expression of known hypoxia responsive genes represented on the UMAP pseudotime trajectory plot and quantified through a linear general additive model over pseudotime. **(b)** Expression of proliferation (*RPS5A*) and expansion markers (*XTH22*) represented on the UMAP pseudotime trajectory plot and quantified through a linear general additive model over pseudotime. Shaded areas in the GAM plot represent Standard Error.

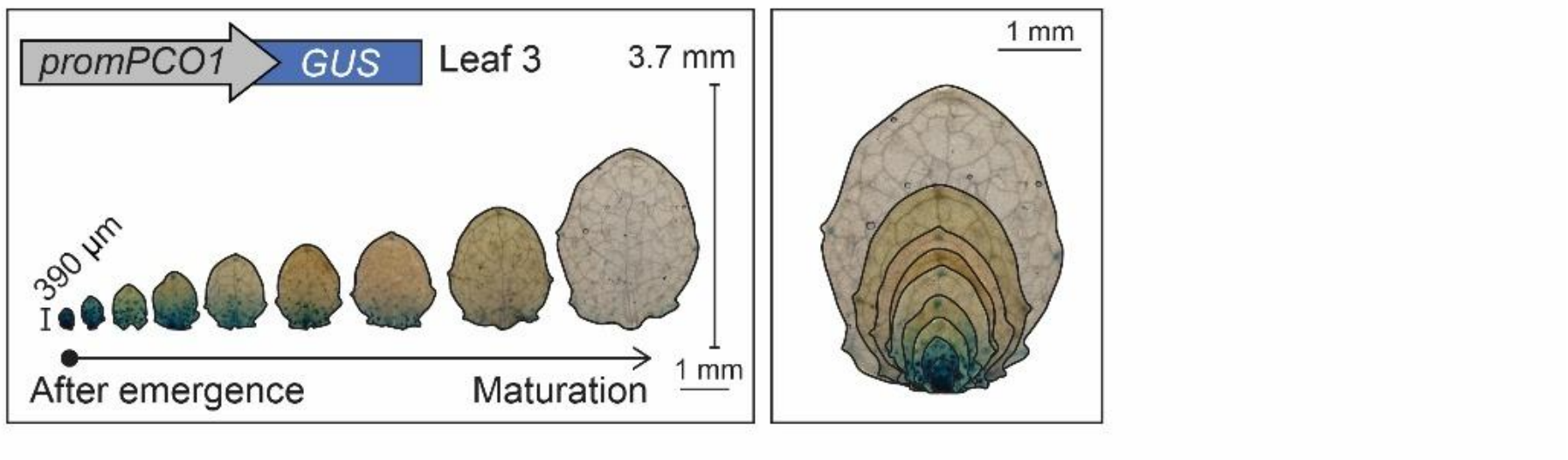

Figure S2. Gradual disappearance of hypoxia signaling in juvenile leaves

GUS staining of leaf 3 of *promPCO1:GUS* at different stages of development.

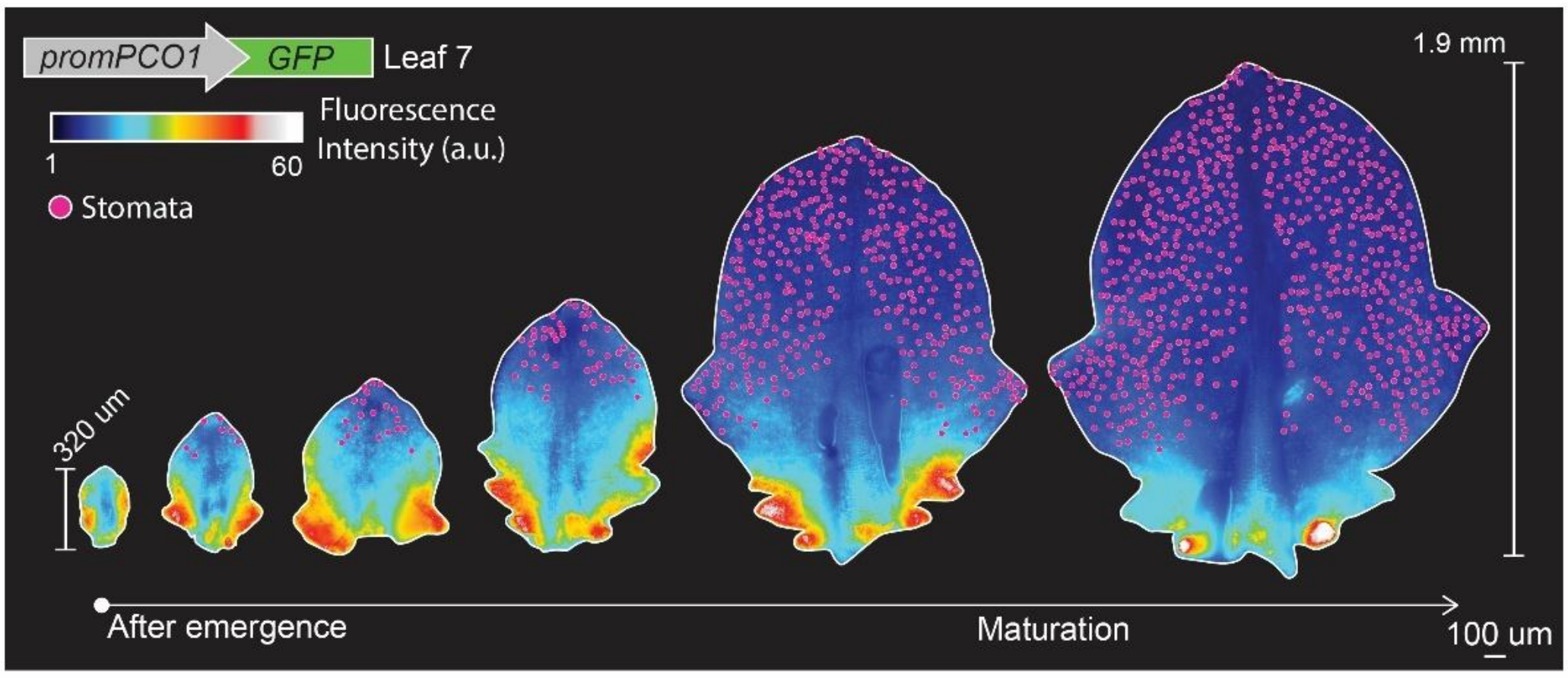

Figure S3 Stomata development is associated with the switching off of hypoxia signaling

GFP and stomata quantification in developing leaf 7 of *promPCO1:GFP* reporter line. GFP intensity is colored using royal lut (range 1 to 60). Stomatal complexes were

counted on brightfield images and indicated with magenta circles.

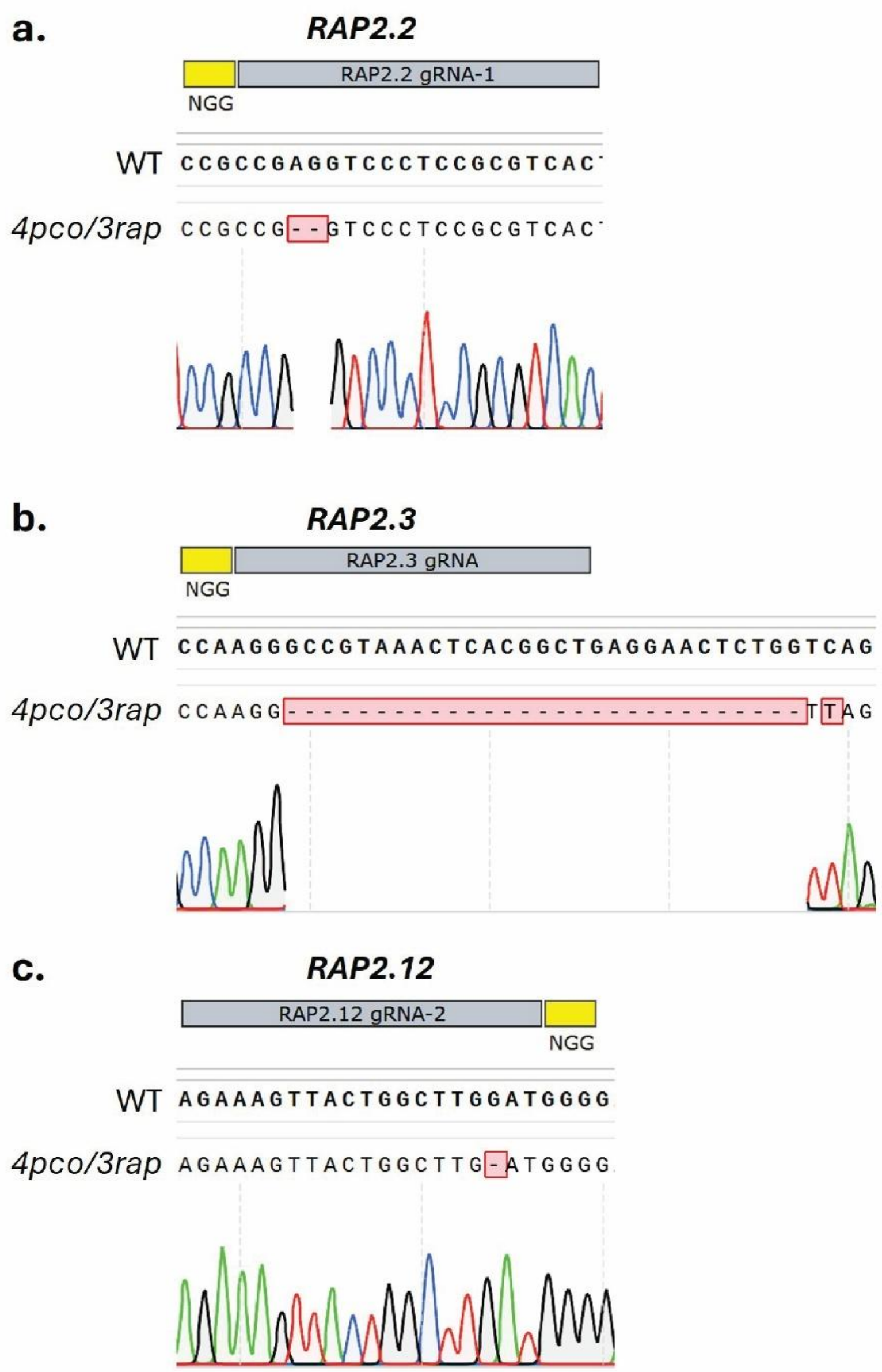

Figure S4. CRISPR/Cas9-induced mutations in RAP2.2, RAP2.3 and RAP2.12 genes

Sequence chromatograms showing representative mutations generated at target sites of RAP2.2, RAP2.3, and RAP2.12 using the indicated gRNAs. **(a)** RAP2.2 gRNA-1: 1-bp deletion within the target sequence. **(b)** RAP2.3 gRNA: large deletion spanning the protospacer region. **(c)** RAP2.12 gRNA-2: 1-bp insertion at the target site. Target sequences are shown above chromatograms, with NGG PAM motifs highlighted. Red boxes indicate the position and type of mutation.

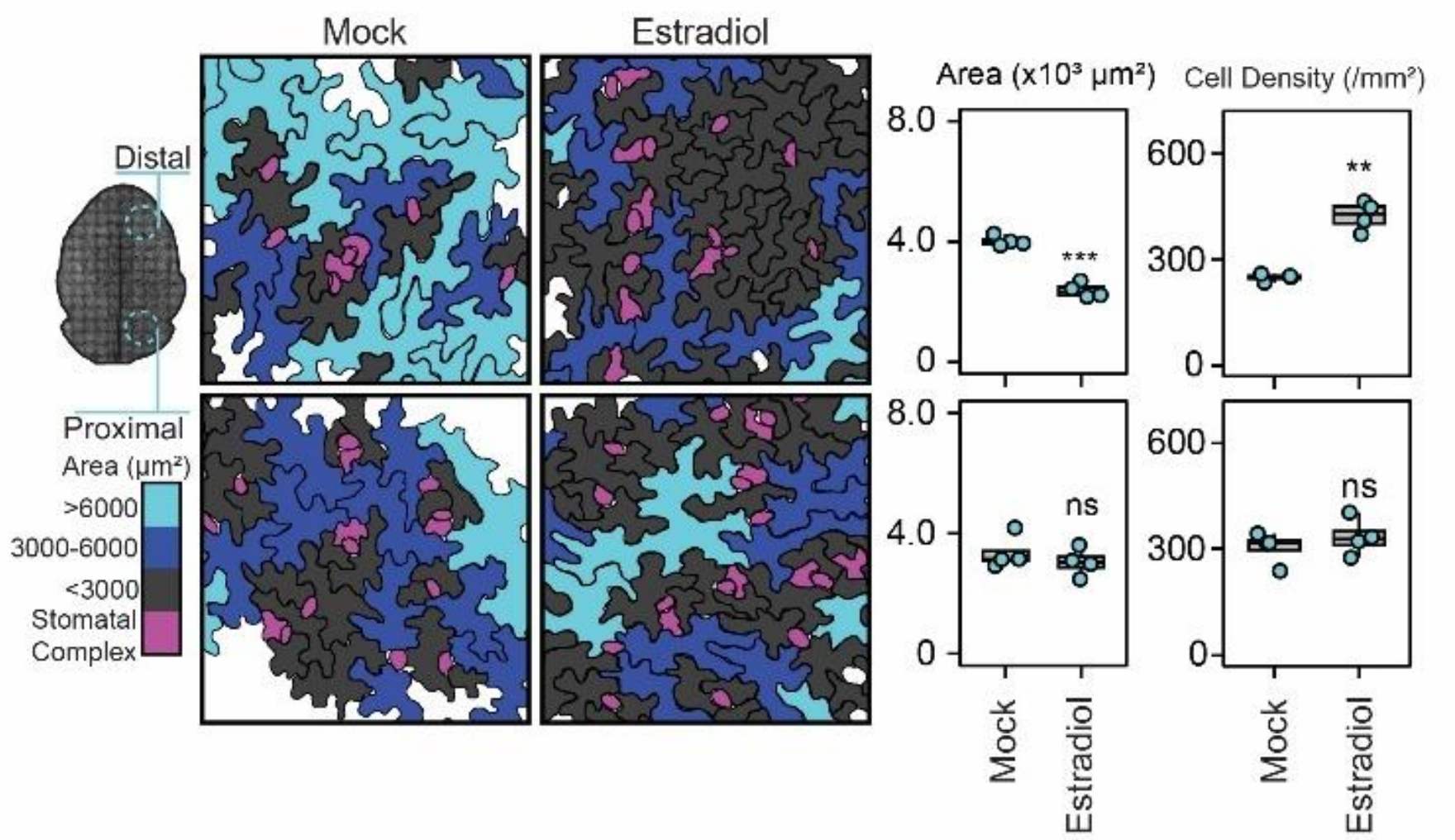

Figure S5 Cellular phenotype resulting from stunting of hypoxia signaling

Segmentation and size quantification of epidermal cells in *RPS5:PCO4* inducible lines. Cells are colored based on area (cyan for cells larger than 6000 µm^2, blue for cells between 3000 and 6000 µm^2, grey for cells smaller than 3000 µm^2). Epidermal cells size and density are also shown as box plots, with each point representing average measurements across one leaf. The images used to indicate distal and proximal leaf sections were reused from Fig. 3c. Statistical differences were assayed via Welsh t-test, with asterisks indicating statically different groups (* p value < 0.05, ** p value < 0.01, *** p value < 0.001).

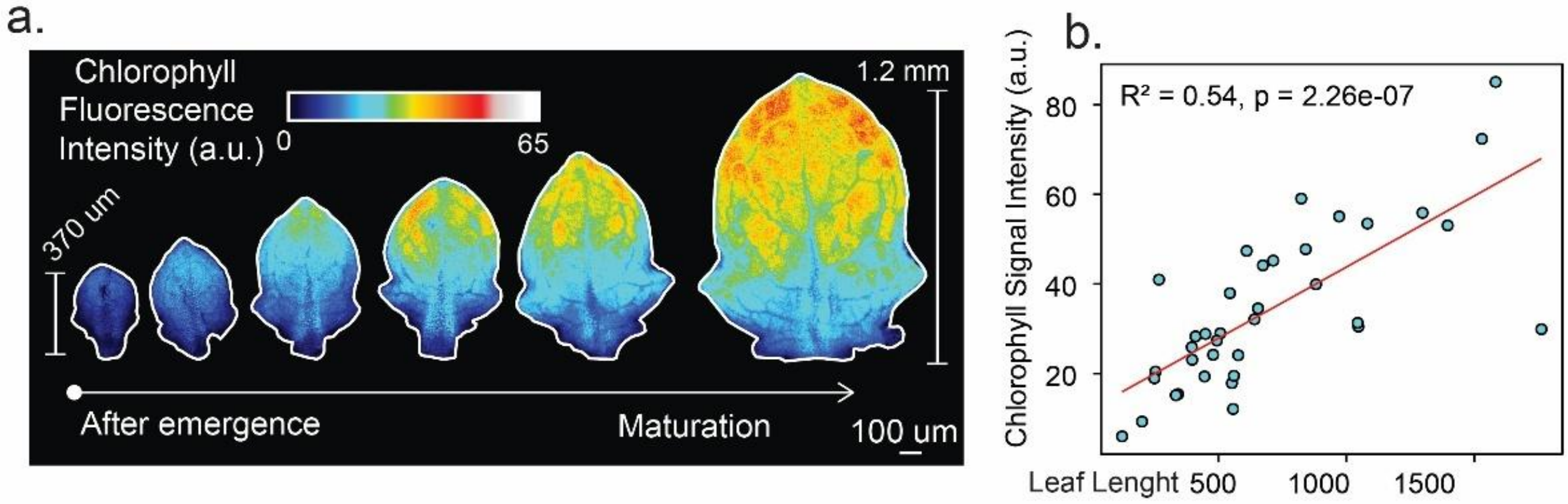

Figure S6. Chlorophyll development over leaf growth in *Arabidopsis thaliana*

(a) Imaging of Chlorophyll autofluorescence across *promPCO1:GFP* developing leaves 7 ± 1 shown in figure 1c. Signal visualized with royal look-up-table. **(b)** Quantification of Chlorophyll autofluorescence in *promPCO1:GFP* developing leaves

in relation to the size of their major axis. Red line represents the linear regression model fitted to the Chlorophyll signal as a function of length. Model fit statistics (R^2 and p-value for the slope) are indicated on the plot.

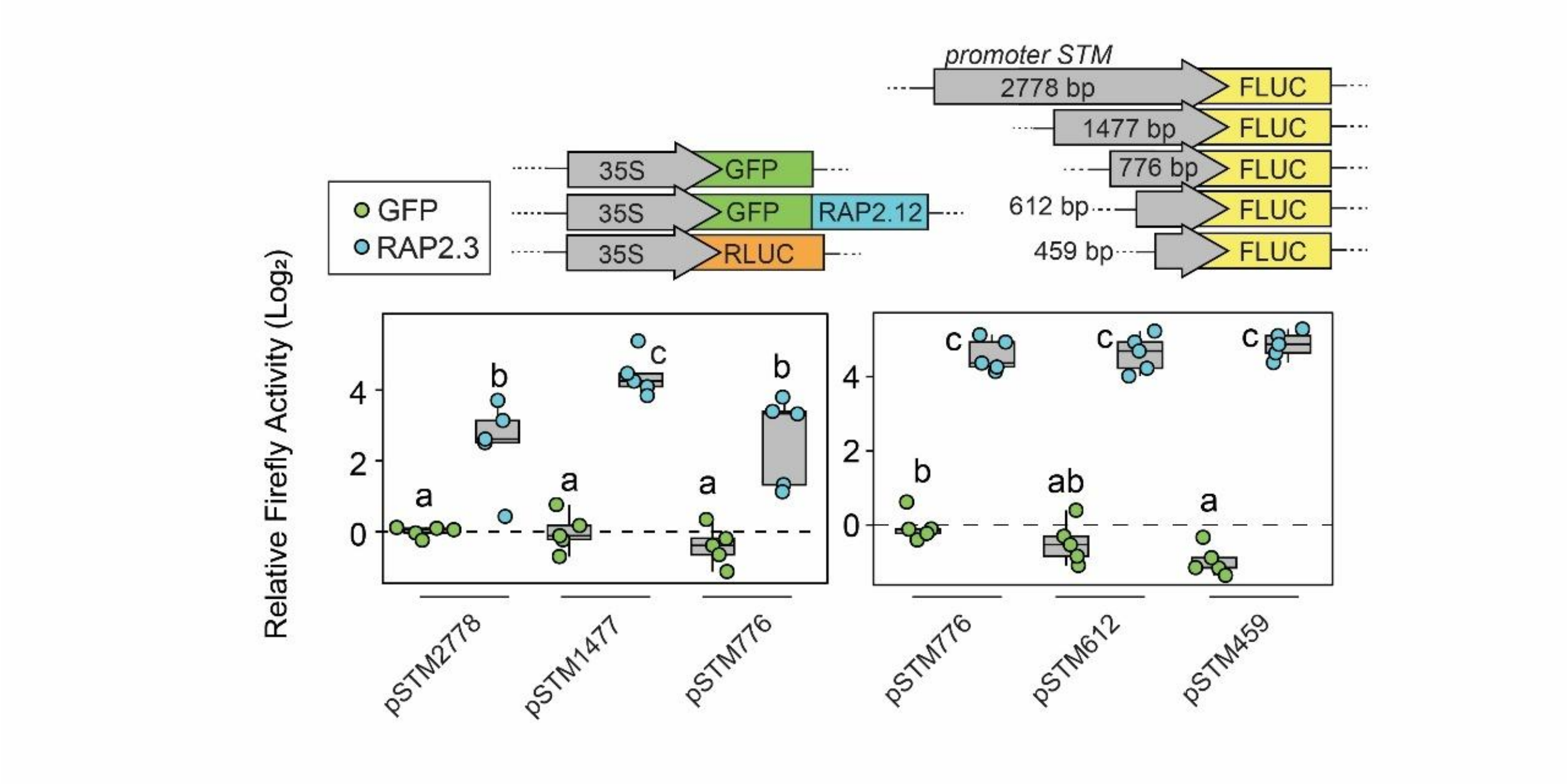

Figure S7. Dissection of the STM promoter region as a target of RAP2.12 activation

Transactivation assay of GFP-RAP2.12 on progressively shorter regions of the *STM* promoter region. Plot shows relative Firefly Luciferase activity normalized to 35S:Renilla luciferase (RLUC). Statistical differences are assayed via two-way Anova and Tuckey post-hoc test for multiple comparisons, with letters indicating statistically different groups (p value < 0.05).

Supplementary Table S1. Primers and guide RNAs used in this study.

Cloning of promoter Regions:

Primer name	5'-3' sequence	AGI-code
pTCL2_Fw_gw	GGGGACAAGTTTGTACAAAAAAGCAGGCTGATGCATTACCAAAATCACTCCACC	at2g30424
pTCL2_Rv_gw	GGGGACCACTTTGTACAAGAAAGCTGGGTCCAGTGGTATTTGAAAGTGAAAGAGG	at2g30424
pMYC4_Fw_gw	GGGGACAAGTTTGTACAAAAAAGCAGGCTGCTTATCATTTTCACCTTTTCTTAAACC	at4g17880
pMYC4_Rv_gw	GGGGACCACTTTGTACAAGAAAGCTGGGTGGAGACGTAACAGTTCTCTGACGTAG	at4g17880
pDEG24Fw	GGGGACAAGTTTGTACAAAAAAGCAGGCTTCGCAACACTAAGCCTACGA	AT5G38420
pDEG24Rv	GGGGACCACTTTGTACAAGAAAGCTGGGTGGAGAACATAGAGGAAGCCAGTAC	AT5G38420
pBAM2Fw	GGGGACAAGTTTGTACAAAAAAGCAGGCTTGTTTGTGTCTCTCCCAATTGA	AT3G49670
pBAM2Rv	GGGGACCACTTTGTACAAGAAAGCTGAGGTTAGCGACGGTGAAGGAATGA	AT3G49670
pPSY1Fw	GGGGACAAGTTTGTACAAAAAAGCAGGCTGCCGGTGGATATGACTCACT	AT5G58650
pPSY1Rv	GGGGACCACTTTGTACAAGAAAGCTGGGTTTGCGGGCTAGAGAAGATGT	AT5G58650
pZFP8Fw	GGGGACAAGTTTGTACAAAAAAGCAGGCTCGTGGACTCGTCATGTGAAG	AT2G41940
pZFP8Rv	GGGGACCACTTTGTACAAGAAAGCTGGGTCTCTCGTCCGTTGGTTTCG	AT2G41940

pSTM2778bp_Fw	CACCCTGGACTTTCCGAGGCACATT	AT1G62360
pSTM459bp	CACCAGAACCCACAGATCACACA	AT1G62360
pSTM_Rv	CTTCTCTTTCTCTCACTAGTA	AT1G62360

Primers for RT-qPCR

**Primer name**	**5'-3' sequence**	**AGI-code**
qTCL2_FW	ACCGCAGAGGTAAATAGTGTGAAA	AT2G30424
qTCL2_RV	TAAATCCCACCTATCACCAACAAG	AT2G30424
qSTI_FW	CGACTTTGCTTCAGCTTGGG	AT2G02480
qSTI_RV	TGGGTCATCGTCAGTTGCTC	AT2G02480
qZFP8_FW	CCACCGACAATCAACGGAAG	AT2G41940
qZFP8_RV	TCATGCGACCTAAACGCTGA	AT2G41940
qPSY1_FW	CTGTTTCCGTTTCAGGTGGG	AT5G58650
qPSY1_RV	GGGTCACCGTAGTCCTCAAC	AT5G58650
qTOP6B_FW	GGTAAGGGGATGCCACATGA	AT3G20780
qTOP6B_RV	CCATCTTTGCACCCAGACCA	AT3G20780
qDEG24_FW	TTCATTGCCTACAAGCCCCC	AT5G38420
qDEG24_RV	ATGGGTTCCAGAATAATCAACGC	AT5G38420
qGL3_FW	GTGTGGTTTCATGGGATATAGGG	AT5G41315
qGL3_RV	CGAACTGAAACTGCGAGGTGT	AT5G41315
qTCL1_FW	AGAAGAGTGGTGGGACGTGA	AT2G30432
qTCL1_RV	ACTTTTGCGTCATTTGTGGGA	AT2G30432
qMYC3_FW	TCATCCCGGTGCTAGGTTCA	AT5G46760
qMYC3_RV	GCTCCCCATCTTCACCGTAG	AT5G46760
qMYC4_FW	GCCGTGTTCTGGCAATCATC	AT4G17880
qMYC4_RV	ACCATCTCCCCAACCTAACAA	AT4G17880
qBAM2_FW2	ACGTGACTGAGAAAGCTCCG	AT3G49670
qBAM2_RV2	CAAACCCAATCGCCGGAAAG	AT3G49670
qSTM_FW	ACCTTCCTCTTTCTCCGGTTATGG	AT1G62360
qSTM_RV	GCGCAAGAGCTGTCCTTTAAGC	AT1G62360
qUBQ10_F	GGCCTTGTATAATCCCTGATGAATAAG	AT4G05320
qUBQ10_R	AAAGAGATAACAGGAACGGAAACATAGT	AT4G05320
qETR2FW	TGTTAGATTCTCCGGCGGCTATG	AT3G23150
qETR2REV	TTCCCATGAATCAACTGCACCAC	AT3G23150
qHUP40 _FWD	GAAACTTGAGTGCGAGTGTG	AT4G24110
qHUP40 _REV	CTCAAACCCAATCTTTTGCT	AT4G24110
qPDC1FW	GACGCCATTCATAACGGTGAAG	AT4G33070
qPDC1RV	GGATTGGGAGGACGGCTGT	AT4G33070
qADH1FW	TCACTGTTGATAATGTCTACCACCG	AT1G77120
qADH1RV	CATGGCCGAAGATACGTGGA	AT1G77120
qLBD41_FW	TGAAGCGCAAGCTAACGCA	AT3G02550
qLBD41_RV	ATCCCAGGACGAAGGTGATTG	AT3G02550
qHB1_FW	TTTGAGGTGGCCAAGTATGCA	AT2G16060
qHB1_RV	TGATCATAAGCCTGACCCCAA	AT2G16060
qACO1FW	ACCAGTCAGAGATGGTCAAGGC	AT4G35830
qACO1REV	TCATCCATCGTCTTGCTGAGTTCC	AT4G35830
qRAP2.3FW	AACTCACGGCTGAGGAACTCTG	AT3G16770
qRAP2.3REV	ACGTTAACTTGGTTGGTGGGATGG	AT3G16770

Guides for RAP2.2/2.3/2.12 CRISPR-Cas9

RAP2.12_1	*TGCGATGATGATTTCGACGT*	AT1G53910
RAP2.12_2	*AGAAAGTTACTGGCTTGGAT*	AT1G53910
RAP2.2_1	*GTGACGCGGAGGGACCTCGG*	AT3G14230
RAP2.2_2	*ATTTCGAAGCTGATTTCCAA*	AT3G14230
RAP2.3	*AGCCGTGAGTTTACGGCCCT*	AT3G16770

Supplementary Table S2. Description of RPS5:PCO4 Inducible Construct

Full plasmid name:

promRPS5: Ω-XVE-tUB10;pLEXA:Ω-PCO4-mCherry-tUB10-HygR in *pGGZ003* backbone.

Sequences for *promRPS5a* (pGGA012), *Ω viral 5'UTR* (pGGB002), *UBQ10 terminator* (*tUB10*) (pGGE009), *mCherry-linker* (pGGD003) and Hygromycin resistance (*HygR*) (pGGF005) are those described by Lampropoulos et al (*58*) in the Green Gate technology plasmid set. Sequences of custom modules follow.

XVE:
atgaaagcgttaacggccaggcaacaagaggtgtttgatctcatccgtgatcacatcagccagacaggtatgccgccgacgcgtgcggaaa
tcgcgcagcgtttggggttccgttccccaaacgcggctgaagaacatctgaaggcgctggcacgcaaaggcgttattgaaattgtttccggcgc
atcacgcgggattcgtctgttgcaggaagaggaagaagggttgccgctggtaggtcgtgtggctgccggtgaaccgtcgagcgcccccccga
ccgatgtcagcctgggggacgagctccacttagacggcgaggacgtggcgatggcgcatgccgacgcgctagacgatttcgatctggacat
gttgggggacggggattccccgggtccgggatttacccccccacgactccgcccccctacggcgctctggatatggccgacttcgagtttgagca
gatgtttaccgatgcccttggaattgacgagtacggtggggatccgtctgctggagacatgagagctgccaacctttggccaagcccgctcatg
atcaaacgctctaagaagaacagcctggccttgtccctgacggccgaccagatggtcagtgccttgttggatgctgagccccccatactctattc
cgagtatgatcctaccagacccttcagtgaagcttcgatgatgggcttactgaccaacctggcagacagggagctggttcacatgatcaactgg
gcgaagagggtgccaggctttgtggatttgaccctccatgatcaggtccaccttctagaatgtgcctggctagagatcctgatgattggactcgtc
tggcgctccatggagcacccagggaagctactgtttgctcctaacttgctcttggacaggaaccagggaaaatgtgtagagggcatggtggag
atcttcgacatgctgctggctacatcatctcggttccgcatgatgaatctgcagggagaggagtttgtgtgcctcaaatctattattttgcttaattctg
gagtgtacacatttctgtccagcaccctgaagtctctggaagagaaggaccatatccaccgagtcctggacaagatcacagacactttgatcc
acctgatggccaaggcaggcctgaccctgcagcagcagcaccagcggctggcccagctcctcctcatcctctcccacatcaggcacatgag
taacaaaggcatggagcatctgtacagcatgaagtgcaagaacgtggtgcccctctatgacctgctgctggagatgctggacgcccaccgcc
tacatgcgcccactagccgtggaggggcatccgtggaggagacggaccaaagccacttggccactgcgggctctacttcatcgcattccttgc
aaaagtattacatcacgggggaggcagagggtttccctgccacagtctga

pLEXA:
agcttgggctgcaggtcgaggctaaaaaactaatcgcattatcatcccctcgacgtactgtacatataaccactggttttatatacagcagtactg
tacatataaccactggttttatatacagcagtcgacgtactgtacatataaccactggttttatatacagcagtactgtacatataaccactggttttat
atacagcagtcgaggtaagattagatatggatatgtatatggatatgtatatggtggtaatgccatgtaatatgctcgactctaggatcttcgcaag
acccttcctctatataaggaagttcatttcatttggagaggacacgctgaagctagtc

PCO4:
atgccttactttgctcagaggctttacaatacttgcaaggcgtctttctcttcagacggaccaataacagaagatgctctggagaaggttcgcaat
gtcttggagaaaatcaagccatctgatgttggaattgagcaggatgctcaattggcacggtccaggtctggtcctctcaatgaacgcaatggaa
gtaatcagtctcccccagcaataaagtatcttcatttgcatgagtgtgacagtttctctataggaatcttctgtatgccaccttcttctatgatacctcttc
ataaccatccgggcatgaccgtgctaagcaagctcgtttatggttcaatgcatgtgaagtcatatgattggctagagcctcaactgaccgaacc
agaggatccatcacaagcaagacctgctaaactggtgaaggatactgagatgacggctcaaagcccagtaacaacattatatccgaaaagt
ggtggcaacattcactgtttcaaagccatcacccattgtgctattcttgacatcttagctccaccttactcttcagagcatgatcggcattgcacttact
tccggaaatccagaagagaagacttaccaggtgaattagaagtggatggagaagtggtcacagacgtgacatggcttgaggaatttcaacc
gcctgatgactttgtgatacgacgaattccgtacagaggtcctgtcattagaacttga

Supplementary Table S3. RNA-Seq annotated data for *4pco* and wildtype of leaf 7 stages 1, 2 and 3.

Supplementary Table S4. GO Terms used for Transcriptome Investigation.

GO ID	GO Term
GO:19827	stem cell population maintenance
GO:9934	regulation of meristem structural organization
GO:90436	leaf pavement cell development
GO:1905622	negative regulation of leaf development
GO:1905623	positive regulation of leaf development
GO:2000024	regulation of leaf development
GO:2000039	regulation of trichome morphogenesis
GO:9965	leaf morphogenesis
GO:10358	leaf shaping
GO:60777	compound leaf morphogenesis
GO:60776	simple leaf morphogenesis
GO:10374	stomatal complex development
GO:2000122	negative regulation of stomatal complex development
GO:2000123	positive regulation of stomatal complex development
GO:10103	stomatal complex morphogenesis
GO:10052	guard cell differentiation
GO:10377	guard cell fate commitment
GO:10441	guard cell development
GO:10376	stomatal complex formation
GO:10440	stomatal lineage progression
GO:10443	meristemoid mother cell division
GO:10444	guard mother cell differentiation
GO:10235	guard mother cell cytokinesis
GO:2000038	regulation of stomatal complex development
GO:48856	anatomical structure development
GO:90558	plant epidermis development
GO:10090	trichome morphogenesis
GO:10026	trichome differentiation
GO:902	cell morphogenesis
GO:90626	plant epidermis morphogenesis
GO:30154	cell differentiation
GO:32502	developmental process
GO:9658	chloroplast organization
GO:15995	chlorophyll biosynthetic process
GO:33305	chlorophyll a biosynthetic process
GO:36067	light-dependent chlorophyll biosynthetic process
GO:36068	light-independent chlorophyll biosynthetic process
GO:10380	regulation of chlorophyll biosynthetic process
GO:1902325	negative regulation of chlorophyll biosynthetic process

Supplementary Table S5. Apex RNA-Seq annotated data for *4pco* and wildtype shoot apices.
